# Thymoquinone Radiosensitizes Human Colorectal Cancer Cells in 2D and 3D Culture Models

**DOI:** 10.3390/cancers14061363

**Published:** 2022-03-08

**Authors:** Samar Al Bitar, Farah Ballout, Alissar Monzer, Mariam Kanso, Nour Saheb, Deborah Mukherji, Walid Faraj, Ayman Tawil, Samer Doughan, Maher Hussein, Wassim Abou-Kheir, Hala Gali-Muhtasib

**Affiliations:** 1 Department of Biology, American University of Beirut, Beirut 1107-2020, Lebanon; sfa28@mail.aub.edu (S.A.B.); frb03@mail.aub.edu (F.B.); aam48@mail.aub.edu (A.M.); 2 Division of General Surgery, Department of Surgery, American University of Beirut Medical Center, Beirut 1107-2020, Lebanon; mk212@aub.edu.lb (M.K.); wf07@aub.edu.lb (W.F.); sd65@aub.edu.lb (S.D.); mh45@aub.edu.lb (M.H.); 3 Department of Pathology and Laboratory Medicine, American University of Beirut Medical Center, Beirut 1107-2020, Lebanon; ns141@aub.edu.lb (N.S.); at04@aub.edu.lb (A.T.); 4 Division of Hematology/Oncology, Department of Internal Medicine, Faculty of Medicine, American University of Beirut Medical Center, Beirut 1107-2020, Lebanon; dm25@aub.edu.lb; 5 Department of Anatomy, Cell Biology and Physiological Sciences, Faculty of Medicine, American University of Beirut, Beirut 1107-2020, Lebanon

**Keywords:** colorectal cancer, cancer stem cells, patient-derived organoids, colon spheres, radiosensitization, DNA repair

## Abstract

**Simple Summary:**

Radiotherapy is a standard of care therapy that kills cancer cells but has limited efficacy in targeting the resistant cancer stem cells (CSCs). The use of radiosensitizers in experimental studies and clinical practice is a successful strategy for eradicating CSCs. Here, we investigated the radiosensitizing potential of thymoquinone (TQ), a natural compound with known anti-cancer activity, in 2D and 3D cultures of colorectal cancer, and in patient-derived organoids. We show that TQ sensitized cancer cells and stem/progenitor cells to radiation mainly through the inhibition of cell survival, DNA repair, and stemness in addition to regulating major pathways implicated in this process. Thus, TQ could be used as a sensitizer to effectively target and kill colorectal cancer cells.

**Abstract:**

Resistance of cancer cells and normal tissue toxicity of ionizing radiation (IR) are known to limit the success of radiotherapy. There is growing interest in using IR with natural compounds to sensitize cancer cells and spare healthy tissues. Thymoquinone (TQ) was shown to radiosensitize several cancers, yet no studies have investigated its radiosensitizing effects on colorectal cancer (CRC). Here, we combined TQ with IR and determined its effects in two-dimensional (2D) and three-dimensional (3D) culture models derived from HCT116 and HT29 CRC cells, and in patient-derived organoids (PDOs). TQ sensitized CRC cells to IR and reduced cell viability and clonogenic survival and was non-toxic to non-tumorigenic intestinal cells. TQ sensitizing effects were associated with G2/M arrest and DNA damage as well as changes in key signaling molecules involved in this process. Combining a low dose of TQ (3 µM) with IR (2 Gy) inhibited sphere formation by 100% at generation 5 and this was associated with inhibition of stemness and DNA repair. These doses also led to ~1.4- to ~3.4-fold decrease in organoid forming ability of PDOs. Our findings show that combining TQ and IR could be a promising therapeutic strategy for eradicating CRC cells.

## 1. Introduction

Radiotherapy is the standard therapy for many cancers; however, its success is often limited by the resistance of some tumor cells and the deleterious effects it causes to surrounding normal tissues [1]. Although high doses of ionizing radiation (IR) may be effective in eradicating tumor cells, they are toxic to normal cells. Evidence has shown that tumor resistance, disease recurrence, and poor prognosis are the result of a small subpopulation of cancer cells, known as cancer stem cells (CSCs) or tumor-initiating cells (TICs) [2,3]. Characteristics of CSCs include self-renewal potential, multi-potency, high tumorigenesis, and activation of proliferative signalling pathways. The latter include Notch, Wnt, and Hedgehog pathways [3,4]. Surviving cells acquire resistance and become less responsive to subsequent radiotherapy cycles through activation of PI3K/Akt, NF-κB, and mammalian target of rapamycin (mTOR) [5].

Several chemotherapeutic drugs have been used in the clinic against colorectal cancer (CRC) either alone or in combination with IR to overcome resistance to radiation and chemotherapy in CSCs. 5-fluorouracil (5-FU), the standard treatment for metastatic CRC, is a pyrimidine analog that inhibits DNA replication thus leading to cell death. 5-FU has been used with IR as a neo-adjuvant therapy for rectal carcinoma to prevent local recurrence [6]. Other drugs have been also studied, validated, and implemented in clinical practices including irinotecan, oxaliplatin, capecitabine, and tegafur [7]. Although these cytotoxic drugs have become standard treatments for patients with CRC, a major challenge remains in the intrinsic and acquired resistance to these therapies, limiting overall survival. Multi drug chemotherapy regimens such as fluorouracil, leucovorin and irinotecan (FOLFIRI); fluorouracil, leucovorin, oxaliplatin and irinotecan (FOLFOXIRI); and oxaliplatin plus fluorouracil and leucovorin (FOLFOX4) have been evaluated in randomized clinical trials. These combination therapies have significantly improved survival of patients with advanced CRC. The addition of monoclonal antibodies for targeted therapy has further improved survival [8,9].

Efforts have aimed to identify natural compounds that could potentially radiosensitize and target cancer cells, while sparing healthy tissues [10]. Thymoquinone (TQ), the bioactive constituent of the black seed oil, exhibits anti-proliferative, anti-oxidant, anti-inflammatory, and apoptotic activities in multiple cancer cell lines and animal models. In addition, TQ inhibits angiogenesis and metastasis of cancer cells [11,12].

TQ has shown promising anti-cancer effects against CRC, when used alone or in combination with chemotherapeutic compounds [13,14,15,16,17,18,19]. In colon adenocarcinoma, TQ was shown to modulate cellular proliferation, migration [13], viability, and apoptosis [16,17].

In animal models, TQ reduced tumor multiplicity and aberrant crypt foci (ACF) and inhibited tumor growth through modulating reactive oxygen species (ROS) and lipid peroxidation levels and reducing dysplasia degree [19], in addition to inducing apoptosis in CRC xenografts [18]. Recently, our group showed that TQ induces apoptosis and DNA damage in 5-FU sensitive and resistant CRC stem/progenitor cells [20]. Combining TQ and 5-FU or using a novel 5-FU/TQ hybrid were more effective than 5-FU alone against resistant CRC stem/progenitor cells by targeting self-renewal capacity and Wnt/ß-Catenin and PI3K/Akt signalling pathways in vitro [20].

Several studies have documented the radiosensitizing role of TQ against cancer with limited toxicity to normal cells. Combining TQ with radiation caused enhanced apoptosis and changes in cell cycle regulation in different cancer models including breast [21,22], head and neck squamous cell carcinoma (HNSCC) [23], and melanoma [24].

In contrast, TQ has shown radioprotective effects in vivo by reducing radiation-induced oxidative [25] and nitrosative stress in brain tissues [26]. Moreover, TQ protected T cells from apoptosis and exhaustion, in gamma radiation-exposed rats [27]. The radioprotective properties of TQ are derived mainly from its ability to act as a free radical scavenger by modulating anti-oxidant enzymes in healthy cells [12], while inducing oxidative stress in cancer cells [12,19].

Although multiple investigators explored the effect of TQ on cancer, no studies have investigated the anti-tumor effect of TQ in combination with IR on CSCs. Here, we studied the radiosensitizing effects of TQ on colorectal CSCs using colonospheres derived from CRC cell lines and patient-derived CRC organoid models. We report the radiosensitization effect of TQ in 2D cultures that lead to inhibition of cell viability, clonogenic survival, and DNA repair. Cell cycle arrest and inhibition of Wnt/β catenin, NF-κB, p-mTOR, and MEK/ERK pathways, in addition to induction of p53 and p21, were associated with this radiosensitization. In 3D culture models, TQ sensitized CSCs to radiation and inhibited stemness and DNA repair mechanisms. TQ radiosensitized CSCs enriched from patient tumor tissues through reducing organoid forming ability and size. This is the first study investigating the effects and the molecular mechanisms of TQ and radiation on CRC stem/progenitor cells and patient-derived organoids (PDOs).

## 2. Materials and Methods

### 2.1. Cell Culture Conditions

Human non-tumorigenic intestinal epithelial FHs74Int cells and the CRC cell lines HCT116, HCT116 p53 null, HT29, and DLD1 were purchased from ATCC (ATCC, Manassas, VA, USA). HCT116, HT29, and DLD1 cells were cultured and maintained in RPMI 1640 (Sigma-Aldrich, Darmstadt, Germany) with 20 mM HEPES and L-Glutamine. HCT116 p53 null cells were cultured in DMEM 4.5 g/L Glucose with L-Glutamine (LONZA, Bornem, Belgium, ANR, Bornem, Belgium). FHs74Int cells were cultured in DMEM 4.5 g/L Glucose with L-Glutamine (LONZA) supplemented with 10 µg/mL insulin and 1% sodium pyruvate. Media was supplemented with antibiotics [1% Penicillin-Streptomycin (100 U/mL)] and 10% heat-inactivated fetal bovine serum (FBS) (Sigma-Aldrich, Darmstadt, Germany). Cells were incubated at 37 °C in a humidified incubator containing 5% CO_2_ and 95% air. All cells were mycoplasma free.

### 2.2. TQ Preparation and Treatment

Directly before use, fresh stocks of the purified synthetic compound TQ (Sigma-Aldrich: CAS: 490-91-5; 99.5% purity) reconstituted in methanol were prepared as per manufacturer’s instructions. Intermediate concentrations were prepared in appropriate media by serial dilutions from stock.

### 2.3. Irradiation

Irradiation of 2D and 3D cultures was performed using the 225 kV biological X-ray Irradiator (Precision X-Ray Inc., Madison, CT, USA) Following treatment with TQ for 24 h, 2D cells were irradiated with 2 Gy once and incubated for another 24 and/or 48 h. For MTT assay, cells were irradiated with different IR doses (1, 2, or 4 Gy). For 3D assays, spheres/organoids were irradiated with 2 Gy once at each generation.

### 2.4. MTT Cell Proliferation Assay

Cell proliferation was determined by MTT ([3-(4,5-dimethylthiazol-2-yl)-2,5-diphenyltetrazolium bromide]) (Sigma-Aldrich) assay as previously described [28]. Briefly, cells were plated in 100 μL complete medium in 96-well culture plates and then treated at 50% confluency in triplicates with various TQ concentrations or IR doses or with TQ followed by irradiation. TQ treatment was replenished every day. At specific time points, MTT (5 mg/mL in DMSO) was added to each well and incubated at 37 °C for 4 h, after which isopropanol was used to dissolve violet crystals. Consequently, MTT optical density (OD) was measured at a wavelength of 595 nm using ELISA reader (Multiskan Ex, Thermo Fisher Scientific, Waltham, MA, USA). Cell proliferation was expressed as a percentage of the control. The percentage of proliferating cells was calculated as: % proliferation = OD of treated cells/OD of untreated cells × 100. GraphPad Prism 6 (version 6.01) Software was used to plot dose response curves and determine IC50s.

### 2.5. Trypan Blue Exclusion Assay

This assay was performed as described previously [28]. Briefly, cells were seeded in 24-well plates and treated accordingly. TQ treatment was replenished every day. Following treatment attached live cells were harvested by trypsin/EDTA and the cell pellet was re-suspended in media. A volume of 50 µL of cell suspension was mixed with 50 µL of trypan blue and live cells were counted using a hemocytometer.

### 2.6. Clonogenic Survival Assay

Cells were seeded in 12-well plates and treated at 50% confluency with TQ alone, radiation alone, or combinations for 48 h. Cells were then trypsinized, counted, and plated at low density (2000–3500 cells) in 100 mm tissue culture dishes and left for 8–10 days in the incubator. Subsequently, cells were washed with PBS, fixed with 95% ethanol, and stained with 1 mL of aqueous 0.5% solution of crystal violet. Colonies having more than 50 cells were counted. The plating efficiency (PE), defined as the ability of control cells to survive and grow into colonies, was calculated as: PE = colonies counted in control/plating density of control. Surviving fraction (SF) for each treatment was calculated as: SF = colonies counted/[cells plated × (PE/100)]. The SF value of each treatment was then plotted.

### 2.7. Cell Cycle Analysis

Cells were seeded in 6-well plates at appropriate densities and treated at 50% confluency with TQ alone, radiation alone or combinations. Dead and live cells were then collected, washed, and incubated in 70% cold ethanol for 30 min. Cells were then incubated for 30 min at 37 °C with 100 μL of propidium iodide (PI) solution [6 μL RNase, 30 μL PI (1 mg/mL) in PBS]. Cell cycle analysis was performed by flow cytometry using Guava EasyCyte8 Flow Cytometer-Millipore. GuavaSoft™ 2.7 Software was used to analyze the distribution of cells in the different phases of the cell cycle.

### 2.8. Sphere Formation Assay

The sphere formation assay was used as previously reported by our group [29]. In brief, single cell suspensions (2000 cells/well) were seeded in cold growth factor-reduced Matrigel™/serum-free RPMI-1640 (1:1) in a total volume of 50 μL. The solution was then plated gently around the rim of individual wells of 24-well culture plates (50 μL per well). Each experimental condition was performed in duplicate. The Matrigel™ (Corning Life Sciences, Corning, NY, USA) was allowed to solidify for 1 h at 37 °C in a humidified incubator. Wells were randomly assigned to control and treatment conditions, and 1 mL/well of complete media (+5% FBS), with or without treatment, was gently added to the center of each well and changed regularly every 2 to 3 days. Irradiation (2 Gy) was performed at day 4 of sphere culture. Sphere counts were performed at day 10–12 of culture. The sphere-forming unit (SFU) was calculated as the ratio of the number of spheres formed/number of cells originally seeded ^x^ 100. Bright field images of the spheres were obtained using Axiovert microscope from Zeiss (San Diego, CA, USA) at 10× magnification.

### 2.9. Propagation Assay

To enrich for the stem-like population of cells, the media was aspirated from the well and the Matrigel^TM^ -containing spheres was collected using ice-cold media. The resulting sphere suspension was centrifuged, and the pellet resuspended with Trypsin/EDTA at 37 °C for 2–2.5 min. Single cells resulting from the dissociation of spheres were counted and re-plated at the same density of 2000 cells/well in 24-well plates as previously described.

### 2.10. Patient-Derived Organoids

#### 2.10.1. Study Design and Ethical Considerations

Human colorectal/rectal tissues were obtained from the American University of Beirut medical center (AUBMC) after obtaining informed consent forms from patients prior to sample acquisition. The study was conducted under the Institutional Review Board (IRB) approvals of the American University of Beirut (AUB) and AUBMC. The work was carried out in accordance with relevant guidelines and regulations, and in agreement with all ethical considerations of the IRB. Tumor tissues and unaffected adjacent tissues were isolated from resected colorectal/rectal segments from patients diagnosed with colorectal/rectal cancer and undergoing colectomy at AUBMC.

#### 2.10.2. Establishment and Propagation of Patient-Derived Colorectal Organoids

Tissues were processed using protocols described by Boehnke K. et al. [30]. Tissues from patients were rinsed with Hank’s Balanced Salt Solution (Gibco, Waltham, MA, USA), minced using sterile scalpels, and digested in adDMEM/F12 (Gibco) supplemented with dihydrochloride kinase inhibitor, Y-27632, 1% P/S, collagenase IV (Sigma-Aldrich, Darmstadt, Germany), and amphotericin B (Sigma-Aldrich, Darmstadt, Germany) at 37 °C for 60 min. During incubation, tissue fragments were mechanically dissociated by repetitive pipetting. The suspension was filtered through a 40 µm cell strainer (Corning) to remove undigested fragments. Isolated single cells were seeded in 24-well plates with Matrigel in a 9:1 ratio at a cell density of 20,000 cells/well. A volume of 20 µL was plated in the middle of the well. Plates were placed upside down in the incubator for 30 min to allow Matrigel to solidify. Cells were cultured with adDMEM/F12 with various factors added to maintain tumor’s biological traits and growth activity. Medium (supplemented with Y-27632) without treatment was changed every 2–3 days. Organoids were propagated at day 10–12. Ice cold adDMEM/F12 medium without factors was added to detach and collect Matrigel with organoids. Organoids were then pelleted and dissociated enzymatically using TrypLE on a shaking platform for 5 min at 37 °C. TrypLE was inactivated by adding adDMEM/F12 containing 5% FBS. Following centrifugation, cells were resuspended in adDMEM/F12 and centrifuged again. Finally, pellet was resuspended in Matrigel and cells plated as described above. Medium with or without TQ was refreshed every 2–3 days and organoids were irradiated at day 4. Organoids were counted at day 10–12 of passage under inverted microscope at 10× magnification. Images were taken and analyzed by Carl Zeiss Zen 3.1.0.0000 image software to determine size. OFC was reported as the number of organoids counted.

### 2.11. Cell Line-Derived Organoids

The organoid formation assay was used as previously reported by our laboratory [29] and as described above with minor modifications. In brief, single cell suspensions (5000 cells/well) seeded in cold growth factor-reduced Matrigel™/serum-free advanced advanced Dulbecco’s Modified Eagle Medium/F12 (adDMEM/F12) (Gibco) in a 9:1 ratio in a total volume of 5 μL in the middle of individual wells of 96-well culture plates. Each experimental condition was performed in duplicate. The Matrigel™ (Corning Life Sciences) was allowed to solidify for 1 h at 37 °C in a humidified incubator. Wells were randomly assigned to control and treatment conditions, and 200 µL/well of advanced DMEM/F12 media with several factors, with or without treatment, was gently added to each well and changed regularly every 2 to 3 days. Irradiation (2 Gy) was performed at day 4 of organoid culture. Organoids were counted at day 10–12 of culture. The organoid-forming count (OFC) was calculated as the ratio of the number of organoids formed/number of organoids in untreated group × 100. Bright field images of the organoids were obtained using Axiovert microscope from Zeiss at 10× magnification.

### 2.12. Immunofluorescent Analysis

#### 2.12.1. 2D Cultures

Immunofluorescent staining was performed to assess the mechanisms of TQ radiosensitization, and its effect combined with IR on DNA repair markers and pathways involved in radioresistance in 2D cells. Cells were grown on glass coverslips and treated at 50% confluency with TQ and then exposed to 0 Gy (no IR) or 2 Gy. At the specific time point, media was removed, and cells were washed with 1x PBS. Cells were then fixed with 4% paraformaldehyde (PFA) in PBS for 20 min and permeabilized with 0.5% Triton X-100 in PBS for 30 min at room temperature. Cells were blocked with blocking buffer (0.1% BSA, 0.2% Triton X-100, 0.05% Tween-20, and 10% normal goat serum in PBS) for 1 h at room temperature. Cells were incubated overnight with Gamma H2AX (γH2AX), p-ATM (Ataxia-telangiectasia mutated), ATR (ATM- and Rad3-Related), MEK, and p-mTOR primary antibodies (Table S1). Cells were washed with PBS containing 0.1% Tween-20 and incubated with fluorophore-conjugated secondary antibody for 1 h at room temperature. After washing, cells were mounted with the anti-fade Fluoro-gel II with DAPI. γH2AX foci were visualized and counted using confocal microscope. For the other molecules, fluorescent signals were captured using a Zeiss LSM 710 laser scanning confocal microscope (Zeiss, Oberkochen, Germany), and images were acquired and analyzed using the Zeiss ZEN image software.

#### 2.12.2. 3D Cultures

Immunostaining was performed according to a protocol described previously by Ballout F. et al. [28]. Spheres and organoids were grown then collected with cold media and centrifuged to washout all Matrigel debris. After centrifugation, spheres and organoids were fixed in 4% PFA. After washing, spheres and organoids were permeabilized with 0.5% Triton X-100 and blocked with sphere blocking buffer (0.1% BSA, 0.2% Triton X-100, 0.05% Tween-20, and 10% normal goat serum in PBS) for 2 h at room temperature. Spheres were then incubated overnight at 4 °C with various primary antibodies (CD44, γH2AX, CK8, and CK19; Table S1) prepared in blocking solution. Organoids were stained for CD44 and CK19. Spheres and organoids were then washed and incubated with secondary antibody for 1 h at room temperature. Finally, spheres and organoids were washed and mounted using 5–7 µL anti-fade reagent Fluoro-gel II with DAPI (Abcam, Cambridge, UK). Fluorescent signals were captured using a Zeiss LSM 710 laser scanning confocal microscope (Zeiss, Oberkochen, Germany), and images were acquired and analyzed using the Zeiss ZEN image software.

### 2.13. Immunofluorescence of Embedded Tumor Tissues

Fresh tissues were fixed in 4% PFA at room temperature for 30 min. Serial tissue sections (4 µm) were stained for H&E and analyzed by an expert who was blinded for the treatment groups. Immunofluorescence staining was performed against stem cell marker CK19. Slides were dried, dewaxed in xylene, and rehydrated using a decreasing alcohol series. Antigen retrieval was performed in 10 mM citrate buffer pH 6 followed by blocking with blocking buffer (10% NGS, 0.1%Triton-X, 3% BSA in PBS). Sections were incubated with primary antibody at 4 °C overnight in antibody solution (2% NGS, 0.1% Triton-X, 3% BSA in PBS). After washing, tissue sections were incubated for 2 h with secondary antibodies diluted in PBS containing 2% NGS and 0.1% Triton-X. Mounting was performed using mounting media with DAPI, after which slides were left to dry and then imaged using Zeiss LSM 710 laser scanning confocal microscope (Zeiss, Oberkochen, Germany) at 10×.

### 2.14. Western Blot Analysis

Cells were plated in 100 mm tissue culture dishes and treated with TQ, IR, or combinations and then collected. Spheres were grown with or without treatment in 24-well plates then collected at G1 with cold media and centrifuged to wash out all Matrigel debris. Cellular protein extracts were prepared using RIPA lysis buffer (sc-24948, Santa Cruz, CA, USA). Protein extracts were quantified using the DC Bio-Rad Protein Assay (Bio-Rad Laboratories, Hercules, CA, USA) according to the manufacturer’s protocol. Protein samples were mixed with 5% β-mercaptoethanol and 2X Laemmli Sample Buffer (Bio-Rad, Hercules, CA, USA) for gel electrophoresis. An equal amount of protein lysate was separated on 8%, 10%, or 12% SDS–PAGE for 2 h at 90 V then transferred onto 0.45 μm nitrocellulose membrane (Bio-Rad, Hercules, CA, USA) in transfer buffer for 2 h at 220 mA at 4 °C. Membranes were blocked with 5% skim milk in tris-buffered saline with 0.1% tween 20 (TBST) for 1 h and then incubated overnight at 4 °C with different primary antibodies (Table S2). Membranes were then washed and incubated with the diluted secondary antibody for 1 h at room temperature. Hybridization with GAPDH-HRP (6C5) (1:10,000–20,000, Abnova, Walnut, CA, USA, #MAB5476) coupled antibody was performed for 30 min at room temperature as housekeeping gene. Target proteins were detected using the ECL system (Bio-Rad, Hercules, CA, USA). Images were generated and quantified using ChemiDoc™ Imaging Systems (Bio-Rad, Hercules, CA, USA).

### 2.15. Statistical Analysis

Statistical analysis was performed using GraphPad Prism 6 Software version 6.0.1. Experimental values are expressed as mean ± SEM. Student’s *t*-test was employed for significance and values of *p* < 0.05 was considered significant (* *p* < 0.05; ** *p* < 0.01; *** *p* < 0.001).

## 3. Results

### 3.1. TQ Sensitizes Colorectal Cancer Cells to Radiation and Reduces Their Cell Viability and Colony Forming Ability

Our first objective was to investigate the effects of TQ and IR on CRC cell lines (HCT116, HCT116 p53 null, HT29, and DLD1), along with human non-tumorigenic intestinal cells (FHs74Int), using MTT (Figures S1–S3). The CRC cell lines have different mutations [31] and sensitivity to TQ [32,33]. Treatment with TQ alone, IR alone, or combinations induced a time- and dose-dependent reduction in proliferation of CRC cell lines. IC50 of TQ at 24 h was highest (112 µM) in HT29 cells (Figure S2b) and lowest (61 µM) in relatively sensitive DLD1 cells (Figure S3b). At 48 h, the IC50 of TQ in HCT116 and HT29 cells was 51.73 µM and 99.46 µM, respectively. IR doses of 1 Gy and 2 Gy reduced the proliferation of HCT116 by 6% and 24%, respectively at 48 h (Figure S2a). None of the IR doses applied to HT29 induced a significant reduction in proliferation at the studied time points. Treatment with TQ (40 µM or 60 µM) followed by irradiation significantly reduced the proliferation of HCT116 cells at 48 h; however, this effect was comparable to TQ alone (Figure S2a). A similar effect was observed in HCT116 p53 null and DLD1 cells (Figure S3a,b). In DLD1 (Figure S3b) and HT29 (Figure S2b) cells, combining TQ and IR reduced proliferation in comparison to IR but not TQ alone at TQ concentrations of 60 µM and 120 µM, respectively. Interestingly, TQ was non-toxic to FHs74Int human non-tumorigenic intestinal cells at doses up to 60 µM at 24 h (Figure S1). IR reduced the proliferation of FHs74Int cells at 24 h at IR dose of 4 Gy, and at 48 h at 1, 2, and 4 Gy of IR. Combining TQ with IR had similar inhibitory effects to TQ alone.

**Figure 1:**
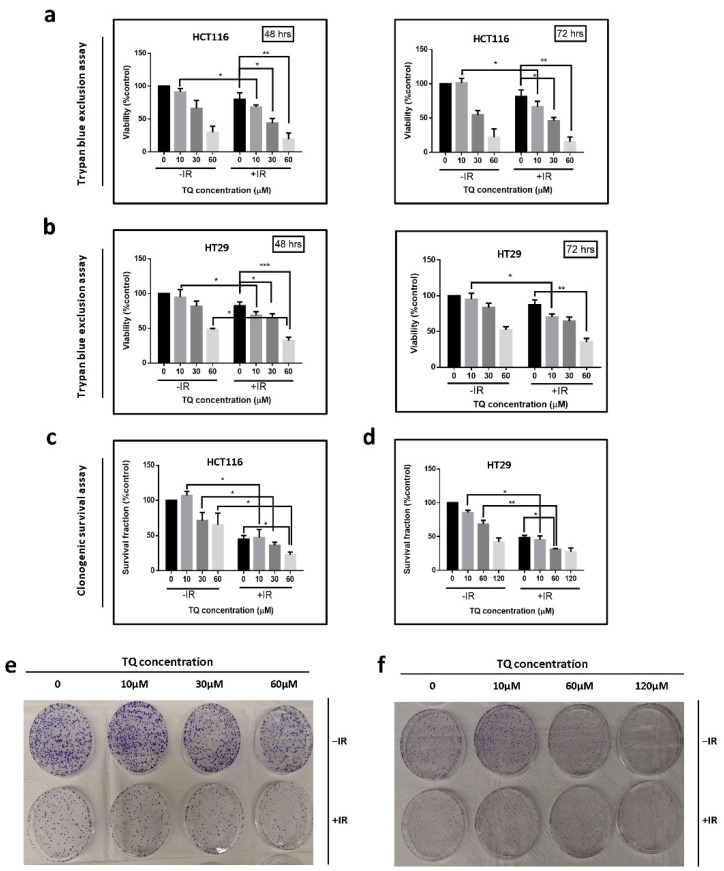
TQ sensitizes colorectal cancer cells to radiation and reduces their cell viability and colony forming ability. (**a**,**b**) HCT116 and HT29 cells were either left untreated or were incubated with TQ alone, IR alone (2 Gy) or combinations for 48 or 72 h. At the specific time point, cell viability was determined using trypan blue exclusion assay. (**c**–**f**) Clonogenic survival assay was used to determine effect of TQ and IR on the long-term survival of HCT116 (**c**) and HT29 (**d**) cells. Cells were treated with TQ, IR, or combinations, after which they were collected and seeded in treatment-free media at low density. After 7–10 days, the resulting colonies were fixed, stained with crystal violet and counted. Representative images of HCT116 (**e**) and HT29 (**f**) colonies are shown. Results are expressed as percentage of the studied group as compared to its control. Data represent an average of three in-dependent experiments. The data are reported as mean ± SEM (* *p* < 0.05; ** *p* < 0.01; *** *p* < 0.001).

We then studied the effect of TQ and IR on the viability and colony formation ability (long-term survival) of HCT116 and HT29 cells using Trypan blue exclusion (Figure 1a,b) and clonogenic survival assays (Figure 1c–f)**.** Cells were treated with TQ alone, IR (2 Gy) alone, or TQ followed by IR. While treatment of HCT116 and HT29 cells with 10 µM TQ alone did not inhibit cell viability at 48 h, combining the same doses of TQ and IR caused significant inhibition of cell viability when compared to TQ alone (Figure 1a,b). A similar effect of TQ and IR combination was observed in HCT116 p53 null and DLD1 cells (Figure S3c,d). Combining 10 µM TQ with 2 Gy IR led to a 32% and 26% inhibition of HCT116 and HT29 cell viability, respectively. A combination of 30 µM TQ and 2 Gy IR led to a significant reduction in HCT116 and HT29 cell viability at 48 h, when compared to IR alone. Importantly, the effect was similar to TQ alone (Figure 1a,b). A similar pattern was observed for the other CRC cell lines treated with TQ and IR combinations (Figure S3c,d). In HT29 cells, combined treatment caused a significant ~60% reduction in cell viability compared to either treatment alone, at 10 µM and 60 µM TQ concentrations (Figure 1b).

For clonogenic survival assay, cells were treated with TQ alone, IR (2 Gy) alone, or TQ followed by IR, after which they were collected, seeded at low densities, allowed to form colonies for 7–10 days, and stained with crystal violet. Combining TQ at concentrations as low as 10 µM with IR had a more pronounced inhibitory effect, when compared to TQ alone (Figure 1c–f). Similar inhibitory effects were observed in the other CRC cell lines under the same conditions (Figure S3e,f). The combination of 60 µM TQ and 2 Gy IR led to 77% and 69% reduction in long-term survival of HCT116 and HT29 cells, respectively, an inhibition that was greater than the effect of either TQ or IR alone (Figure 1c–f). The combination of TQ and IR inhibited HT29 colony formation by 55%, 69%, and 72.6% at 10 µM, 60 µM, and 120 µM TQ, respectively.

### 3.2. TQ Enhances IR-Induced Cell Cycle Arrest at G2/M Phase in Colorectal Cancer Cells

We then determined the effect of combination treatment on cell cycle distribution in HCT116 and HT29 cells using flow cytometry with DNA staining. While IR (2 Gy) alone induced a slight increase in the percentage of HCT116 cells in G2/M phase, combining IR with TQ (10 µM and 30 µM) induced a significant accumulation of cells in G2/M phase (Figure S4a). Moreover, the decrease in percentage of cells at G0/G1 was significant in HCT116 cells treated with 30 µM TQ and IR. In HT29, cell cycle arrest was observed in irradiated cells and in cells treated with 60 µM TQ and was more pronounced in cells treated with TQ and IR combination compared to either treatment alone (Figure S4b). Interestingly, when combined with IR, TQ induced a significant increase in the G2/M population from 21% in the control to 26% and 31% at TQ concentrations of 10 µM and 60 µM, respectively.

### 3.3. TQ Radiosensitization of Colorectal Cancer Cells Is Associated with DNA Repair Inhibition

**Figure 2:**
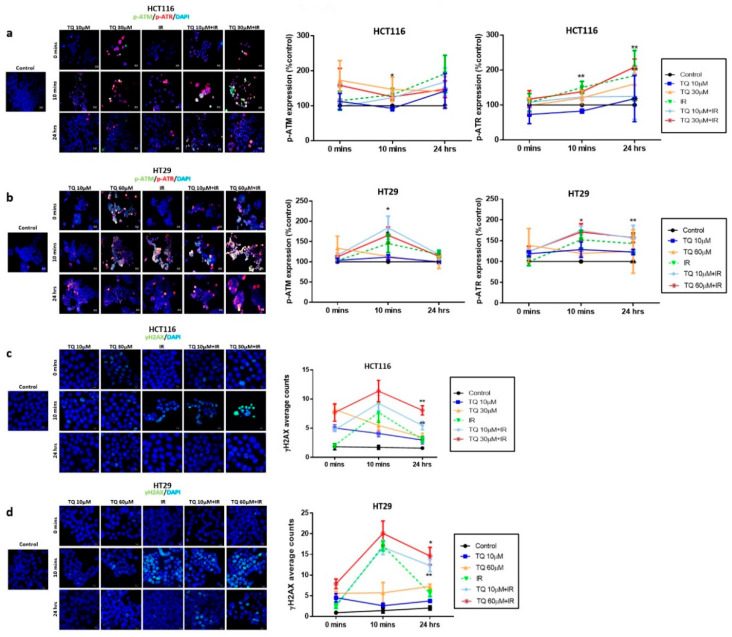
TQ radiosensitization of colorectal cancer cells is associated with DNA repair inhibition. HCT116 and HT29 cells were either left untreated or incubated with TQ for 24 h followed by irradiation at 2 Gy. Cells were then fixed at 0 min, 10 min and 24 h post irradiation, followed by permeabilization and staining for p-ATR, p-ATM (**a**,**b**), and Gamma H2AX (γH2AX) (**c**,**d**). Quantification and representative images are shown. Quantification of p-ATM and p-ATR intensity was performed using Carl Zeiss Zen 2012 image software. γH2AX foci were counted using the confocal microscope. Data represent an average of three independent experiments and are reported as mean ± SEM (* *p* < 0.05; ** *p* < 0.01 significantly different from control for p-ATM and p-ATR, and from IR for γH2AX). Scale bar for immunofluorescent images is 20 µm.

To elucidate the mechanism underlying the observed G2/M arrest in response to TQ and IR, we analyzed the dynamics of γH2AXand the kinases responsible for its phosphorylation during DNA damage response (DDR), mainly ATM and ATR expression (Figure 2). ATM and ATR are members of the phosphatidyl inositol 3-kinase-like family of serine/threonine protein kinases (PIKKs) and are involved in the regulation of G2/M checkpoint. To check whether G2/M arrest is ATM- or ATR-dependent, we analyzed the dynamics of these regulators over a 48 h period (Figure 2a,b). HCT116 and HT29 cells were treated with TQ for 24 h followed by irradiation at 2 Gy. Cells were then fixed at 0 min, 10 min, and 24 h post irradiation, followed by permeabilization and staining for p-ATM and p-ATR and for the DNA damage and repair marker, γ-H2AX. We observed similar activation of ATM and ATR in the two cell lines. Interestingly, combining 30 µM TQ and IR in HCT116 cells led to a significant increase in p-ATM, 10 min after irradiation (Figure 2a). At 24 h, the levels of p-ATM were high in combination-treated HCT116 cells but were not significant compared to the control. p-ATR was upregulated 10 min post irradiation in HCT116 cells treated with IR alone or combination, and this increase was persistent at 24 h, especially in cells treated with combination of 30 µM TQ and IR (for more details check Table S3). In HT29 cells, the increase in p-ATM and p-ATR 10 min post irradiation was insignificant compared to the control; however, when cells were treated with either 10 µM or 60 µM TQ prior to irradiation, the levels of both enzymes significantly increased 10 min after irradiation (Figure 2b, Table S4). Importantly, at 24 h the levels of p-ATM were similar between the HT29 combination-treated cells and control cells. On the other hand, the level of p-ATR remained significantly high in cells treated with 60 µM TQ and IR.

In HCT116 cells, γH2AX expression was upregulated in the presence of 10 µM or 30 µM TQ (Figure 2c). γH2AX foci count in irradiated cells was comparable to cells treated with 30 µM TQ at 10 min after IR. The highest peak of γH2AX was observed at 10 min in cells treated with TQ and IR and its expression remained significantly high 24 h after radiation in cells treated with IR and 10 µM or 30 µM TQ (>2-fold increase) (Figure 2c). Importantly, the levels of γ-H2AX in cells treated with 30 µM TQ and IR were significantly higher than cells treated with TQ alone at 10 min and 24 h post irradiation. In HT29 cells, a similar increase of γH2AX expression was observed (Figure 2d). A dose of 10 µM TQ was sufficient to sensitize these cells to radiation and induce 2-fold increase in γH2AX counts at 24 h post IR. Higher TQ concentrations resulted in a higher accumulation of DNA damage. TQ and IR caused a more pronounced upregulation of γH2AX expression than TQ alone.

### 3.4. TQ Sensitizes Cancer Cells to Radiation through Targeting Major Pathways Implicated in Radiation Therapy

**Figure 3:**
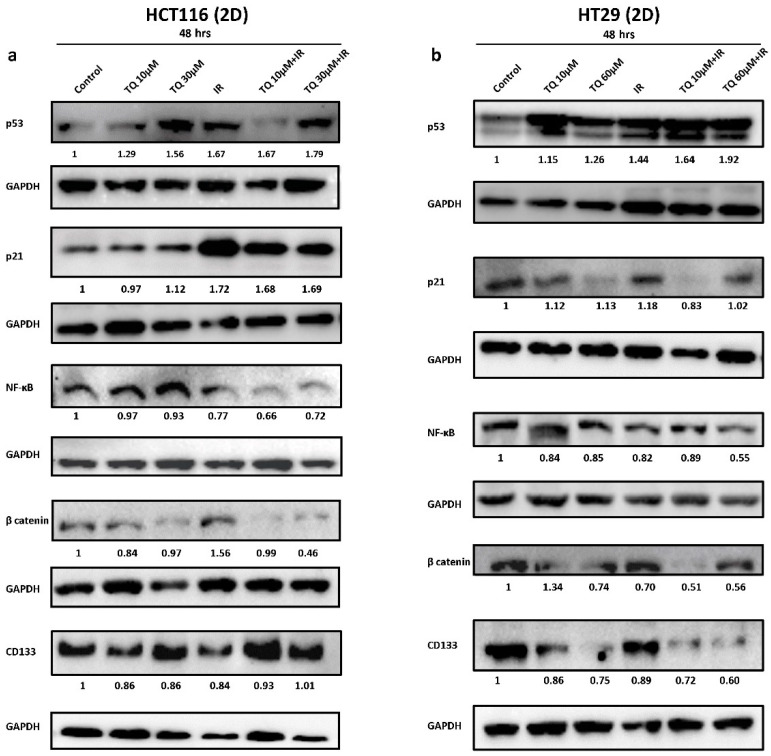
TQ sensitizes colorectal cancer cells to radiation through targeting major pathways implicated in radiation therapy. Western blot analysis of p53, p21, NF-κB (p65), β catenin, and CD133 48 h post treatment with TQ, IR, or TQ+IR in HCT116 (**a**) and HT29 cells (**b**). Fold expression changes normalized to GAPDH. Data represent an average of at least three independent experiments.

To determine the mechanism of TQ radiosensitization, we analyzed the expression of several molecules involved in survival and response to radiation (Figure 3 and Figure S5). In HCT116, combination of 30 µM TQ and IR significantly reduced the expression of p-mTOR; however, this decrease in expression was comparable to TQ alone (Figure S5a). MEK was significantly reduced in combination-treated cells and the observed effect was comparable to TQ or IR alone (Figure S5c). In HT29 cells, combining 10 µM TQ and IR resulted in a significant inhibition of p-mTOR and MEK; however, the reduction was similar to TQ alone (Figure S5b,d). A dose of 60 µM TQ was able to reverse the slight p-mTOR increase induced by IR. In HCT116 cells, Western blot analysis showed that the increase in p53 expression in cells treated with 30 µM TQ and IR was comparable to individual treatments, with slight enhancement in p53 expression in cells treated with combinations (Figure 3a and Figure S9). IR alone and its combination with TQ (10 µM or 30 µM) induced an upregulation in p21 expression by ~1.7-fold. Combining TQ (10 µM or 30 µM) with IR led to a significant reduction in NF-κB expression and combining 30 µM TQ with IR reduced β catenin expression. CD133 levels did not change upon treatment of HCT116 cells. In HT29 cells, p53 was upregulated by more than 1.6-fold in response to TQ (10 µM or 60 µM) and IR, whereas the increase in p21 was insignificant under these conditions (Figure 3b and Figure S10). Importantly, 60 µM TQ and IR significantly reduced the expression of NF-κB and this reduction was significant when compared to IR alone. Notably, combining 60 µM TQ and IR led to a significant reduction in β catenin and CD133 expression.

### 3.5. TQ Radiosensitizes Colorectal Cancer Stem/Progenitor Cells and Reduces Their Sphere-Forming and Self-Renewal Ability

**Figure 4:**
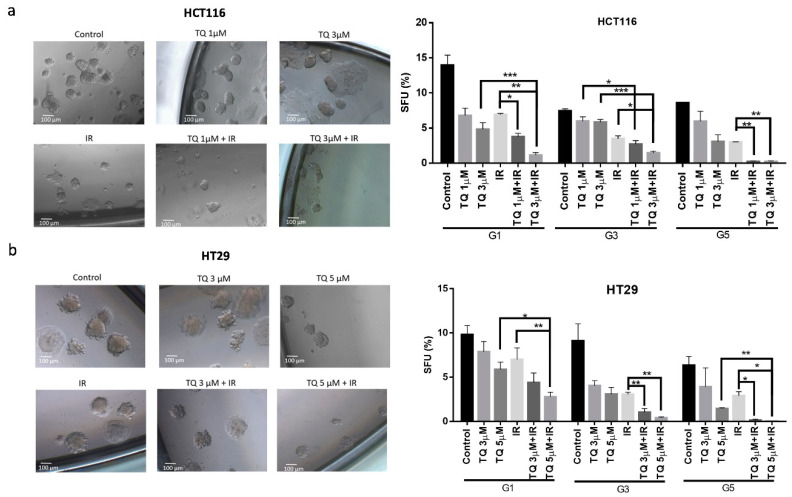
TQ radiosensitizes colorectal cancer stem/progenitor cells and reduces their sphere-forming and self-renewal ability. Sphere forming unit (SFU) obtained from serially passaged colonospheres over five generations is shown for HCT116 (**a**) and HT29 (**b**) spheres treated with TQ (1, 3 and 5 µM), radiation (2 Gy), or combinations. SFU is calculated according to the following formula: SFU = (number of spheres counted ÷ number of input cells) × 100. HCT116 and HT29 cells were suspended in Growth Factor reduced Matrigel/serum-free media (ratio 50:50) and allowed to grow in media with 5%FBS (with or without treatment) to enrich for colorectal CSCs. Generated spheres are referred to as G1 (Generation 1) spheres. After each propagation, cells that were initially treated with TQ, IR, TQ+IR, or media (control) were seeded into separate wells. Spheres were propagated for five generations in duplicates for each condition. Data represent an average of three independent experiments and are reported as mean ± SEM (* *p* < 0.05; ** *p* < 0.01; *** *p* < 0.001). Representative bright-field images showing the effect of TQ, IR, and combinations on SFU of HCT116 and HT29 spheres are shown next to the respective graphs. Images were visualized by Axiovert inverted microscope at 10× magnification and analyzed by Carl Zeiss Zen 2012 image software. Scale bar 100 µm.

Self-renewal is one of the major hallmarks of cancer stem/progenitor cells. To assess the effect of TQ and IR on sphere-forming and self-renewal abilities, cells were seeded with Matrigel in 3D culture sphere formation assay and spheres were propagated till generation 5 (G5). At each generation, cells were treated with TQ and then irradiated at day 4, after which spheres were imaged and counted. At G1, combining TQ (1 µM or 3 µM) with IR significantly reduced the sphere forming ability of HCT116, in comparison to IR alone (Figure 4a). Treatment with 1 µM TQ and IR reduced HCT116 colonospheres by more than 3-fold, whereas treatment with 3 µM TQ and IR decreased the number of spheres by a remarkable 12-fold. At G3, inhibition by 3 µM TQ and IR was persistent and greater than either treatment alone. Notably, at G5, 3 µM TQ and IR led to 97% reduction in sphere count. While treatment of HT29 cells with TQ or IR alone induced no significant decrease in SFU, combining 5 µM TQ and IR led to ~68% decrease at G1 (Figure 4b). Interestingly, successive propagation and treatment of HT29 cells with 3 µM TQ and IR, significantly decreased SFU by ~84% at G3 and by ~100% at G5. This decrease was greater than that of individual treatments.

Interestingly, upon withdrawal of combination treatment in the subsequent generation, HCT116 and HT29 colonospheres did regain some of their sphere-forming ability, yet their counts were still significantly lower than that of the control at the same generation (Figure S6). This indicates that the treatment is partially irreversible.

### 3.6. TQ Radiosensitization of Stem/Progenitor Cells Is Associated with Inhibition of DNA Repair and Stemness

**Figure 5:**
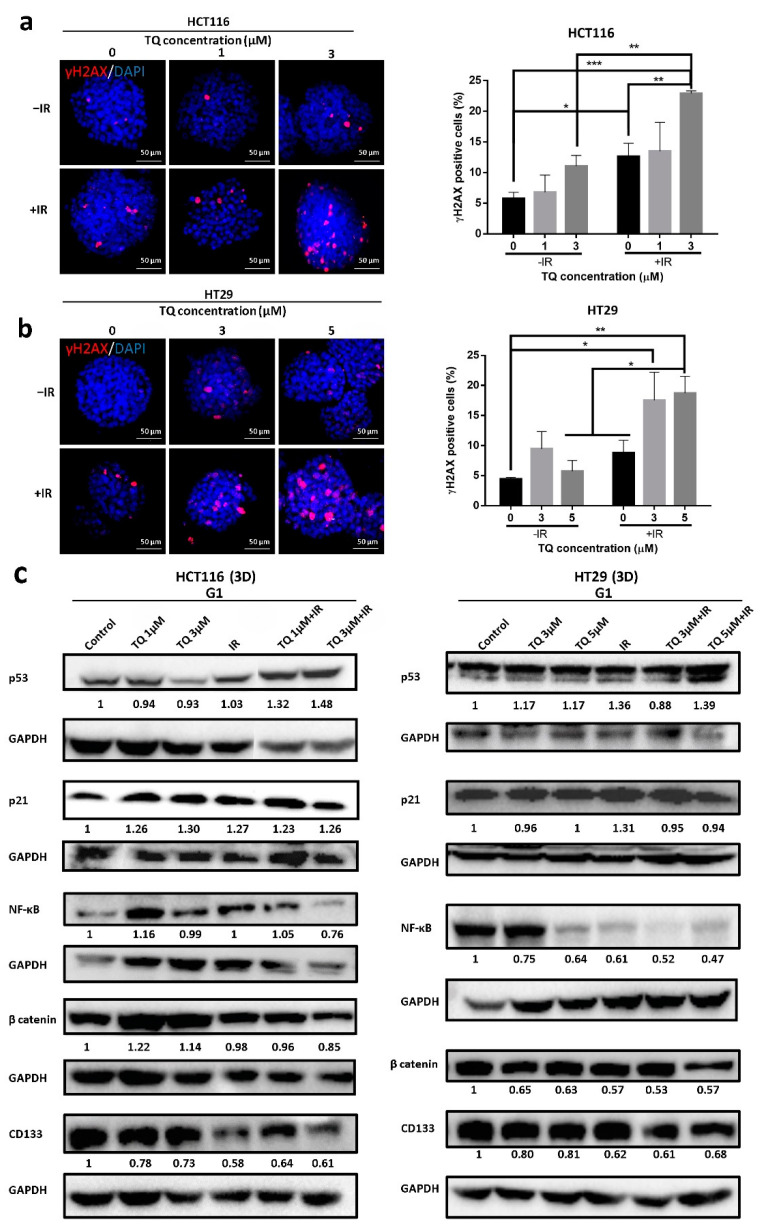
TQ radiosensitization of CRC stem/progenitor cells leads to inhibition of DNA repair and stemness. Representative images of TQ, IR, and combinations treated HCT116 (**a**) and HT29 (**b**) G1 spheres after γH2AX staining. γH2AX positive cells were counted and normalized to size. Data represent an average of three independent experiments and are reported as mean ± SEM (* *p* < 0.05; ** *p* < 0.01; *** *p* < 0.001). Scale bar 50 µm. (**c**) Analysis of p53, p21, NF-κB (p65), β catenin, and CD133 protein expression in HCT116 and HT29 G1 spheres following treatment with TQ, IR, and combinations. Fold expression changes normalized to GAPDH.

We checked for DNA damage in spheres treated with individual or combined treatment and our results indicated that IR alone induced more than 2-fold increase in the γH2AX foci in HCT116 spheres (Figure 5a). Interestingly, combining 3 µM TQ and IR led to a remarkable ~4-fold increase in the γH2AX and this increase was more pronounced than TQ alone and IR alone. In HT29 spheres (Figure 5b), TQ (3 µM or 5 µM) and IR treatment induced a significant ~4-fold upregulation of γH2AX, whereas TQ or IR alone resulted in no significant increase in γH2AX foci, suggesting a delay in DNA damage repair after exposure to combination of TQ and IR.

Analysis of p53 expression by Western blot showed a significant upregulation in HCT116 spheres upon treatment with 3 µM TQ and IR and was significant when compared to TQ alone (Figure 5c and Figure S11). p21 expression was upregulated in HCT116 spheres treated with 3 µM TQ alone and in combination with IR. Combining 3 µM TQ and IR resulted in a significant reduction in NF-κB expression, and the reduction was more pronounced in comparison to either treatment alone. Combining 3 µM TQ and IR reduced the expression levels of β catenin and CD133. In HT29 spheres, a combination of 5 µM TQ and IR led to an increase in p53 but not p21 levels (Figure 5c and Figure S12). Combining 5 µM TQ and IR decreased the expression levels of NF-κB and β catenin by ~2-fold. The combination of TQ (3 µM or 5 µM) with IR reduced the levels of CD133 by more than 1.5-fold. Immunostaining analysis showed that 3 µM TQ alone and the combination of 3 µM TQ and IR decreased expression of CD44, a CRC stem cell marker, in HCT116 spheres by 1.2–1.43-fold (Figure S7a). In HT29 spheres, 5 µM TQ decreased CD44 expression level by ~1.5-fold, whereas combining 5 µM TQ with IR led to a ~1.8-fold reduction (Figure S7b). This reduction was significant when compared to IR alone. In HCT116 spheres, TQ or IR alone had no effect on the expression level of the epithelial marker CK8, while combining 3 µM TQ with IR led to a ~1.5-fold decrease (Figure S7c). Combining TQ (1 µM or 3 µM) with IR significantly reduced the level of the stem cell marker CK19 and in a comparable way to 3 µM TQ alone. In HT29 spheres, none of the treatments induced significant changes in the expression of CK8 or CK19 (Figure S7d), suggesting that the inhibitory mechanism is different in HCT116 and HT29 spheres.

### 3.7. Effect of TQ and IR on Patient-Derived Tumor Organoids

**Figure 6:**
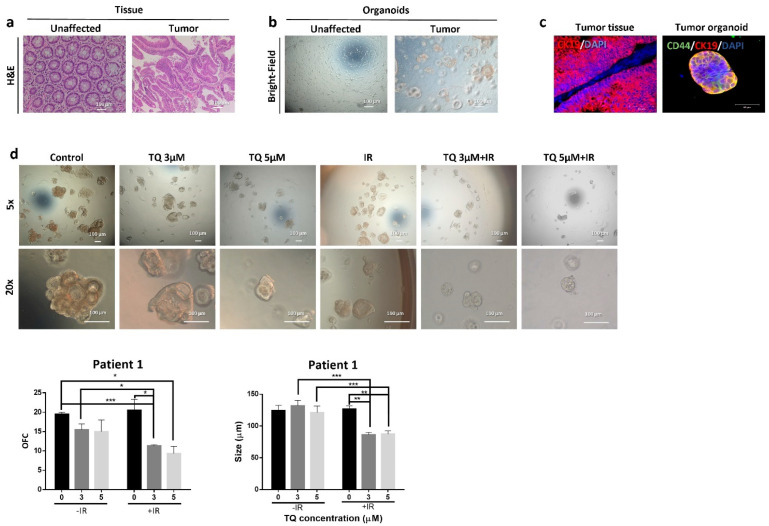
TQ radiosensitizes patient 1-derived rectal cancer organoids and reduces their organoid-forming ability and size. (**a**) Representative images of H&E stain of unaffected rectum and rectal cancer tissue from patient 1. (**b**) Representative bright-field images of organoids derived from unaffected rectum and rectal cancer samples (patient 1). (**c**) Immunofluorescent images of rectal tumor issues and organoids stained for CD44 and CK19. Images were obtained using confocal microscopy. (**d**) Representative bright-field images of organoids derived from rectal cancer patient 1 sample and treated with TQ (3 and 5 µM), radiation (2 Gy), or combinations. Fresh unaffected and tumor tissues were digested, and single cells were resuspended in 90% Growth Factor reduced Matrigel and 10% serum-free colon media and allowed to grow in serum-free colon media (without treatment). Generated organoids are referred to as G1 organoids. Organoids were propagated to G2 and treated with TQ, IR, or combinations. OFC and size were calculated, and average values were reported as mean ± SEM (* *p* < 0.05, ** *p* < 0.01, *** *p* < 0.001). Images were visualized by Axiovert inverted microscope at 10× magnification. Scale bar for bright-field images is 100 µm and for immunofluorescent images is 50 µm.

**Figure 7:**
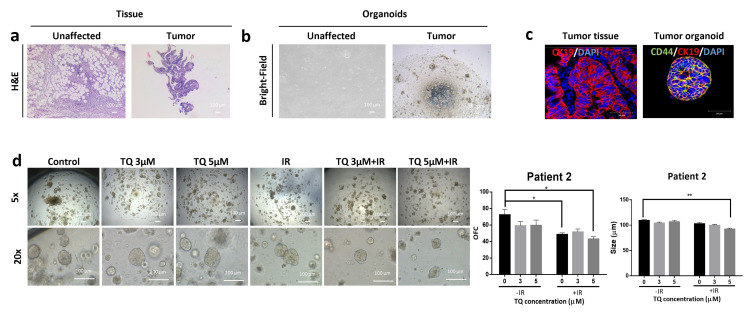
TQ and radiation reduce organoid-forming ability and size of patient 2-derived colon cancer organoids. (**a**) Representative images of H&E stain of unaffected and tumor colon tissue from patient 2. (**b**) Representative bright-field images of organoids derived from unaffected and tumor colon patient samples (patient 2). (**c**) Immunofluorescent images of tumor colon issues and organoids stained for CD44 and CK19. Images were obtained using confocal microscopy. (**d**) Representative bright-field images of organoids derived from tumor colon patient 2 sample and treated with TQ (3 and 5 µM), radiation (2 Gy), or combinations. Fresh unaffected and tumor tissues were digested, and single cells were resuspended in 90% Growth Factor reduced Matrigel and 10% serum-free colon media and allowed to grow in serum-free colon media (without treatment). Generated organoids are referred to as G1 organoids. Organoids were propagated to G4 and treated with TQ, IR, or combination. OFC and size were calculated, and average values were reported as mean ± SEM (* *p* < 0.05, ** *p* < 0.01. Images were visualized by Axiovert inverted microscope at 10× magnification. Scale bar for bright-field images is 100 µm and for immunofluorescent images is 50 µm.

**Figure 8:**
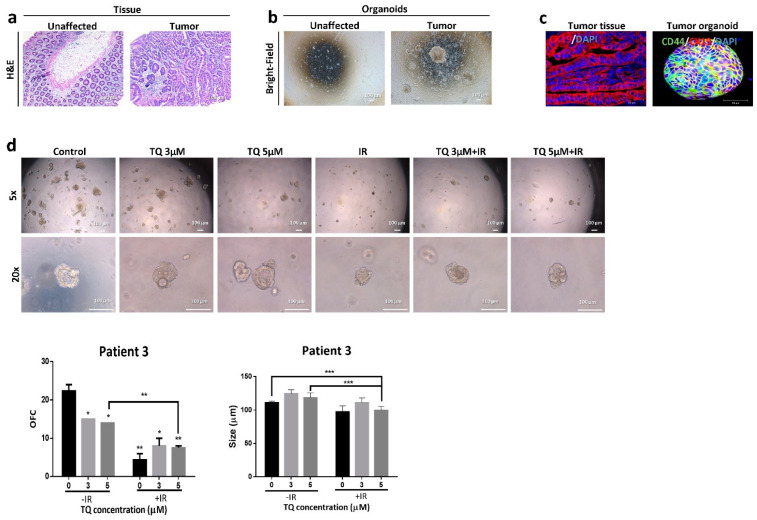
TQ and radiation reduce organoid-forming ability and size of patient 3-derived colon cancer organoids. (**a**) Representative images of H&E stain of unaffected and tumor colon tissue from patient 3. (**b**) Representative bright-field images of organoids derived from unaffected and tumor colon patient samples (patient 3). (**c**) Immunofluorescent images of tumor colon tissues and organoids stained for CD44 and CK19. Images were obtained using confocal microscopy. (**d**) Representative bright-field images of organoids derived from tumor colon patient 3 sample and treated with TQ (3 and 5 µM), radiation (2 Gy), or combinations. Fresh unaffected and tumor tissues were digested, and single cells were resuspended in 90% Growth Factor reduced Matrigel and 10% serum-free colon media and allowed to grow in serum-free colon media (without treatment). Generated organoids are referred to as G1 organoids. Organoids were propagated to G2 and treated with TQ, IR, or combination. OFC and size were calculated, and average values were reported as mean ± SEM (* *p* < 0.05, ** *p* < 0.01, *** *p* < 0.001). Images were visualized by Axiovert inverted microscope at 10× magnification. Scale bar for bright-field images is 100 µm and for immunofluorescent images is 50 µm.

We succeeded in establishing PDOs and propagating them to model CRC disease in 3D culture. Fresh unaffected and tumor tissues were processed as mentioned before; however, only tumor samples successfully formed organoids in culture (Figure 6b, Figure 7b and Figure 8b). Following propagation, tumor organoids were either treated with TQ (3 µM or 5 µM), IR (2 Gy), or combinations. After 10–12 days, the number and size of organoids were calculated. While IR or TQ alone had no significant effect on patient 1 organoid count or size, combining TQ (3 µM or 5 µM) and IR significantly reduced both organoid count and size (Figure 6d). There was ~1.7- fold and ~2-fold reduction in OFC upon treatment with TQ and IR at TQ concentrations of 3 µM and 5 µM, respectively. The average organoid size was significantly reduced by ~1.4-fold in the combined treatment compared to either treatment alone. Interestingly, organoids established from the HT29 cells and treated with TQ (3 µM or 5 µM), IR (2 Gy), or combinations showed similar response to organoids from patient 1 (Figure S8a). There was no significant effect of IR alone on HT29 OFC, whereas 5 µM TQ alone led to ~13% reduction. Interestingly, combining 5 µM TQ with IR resulted in a 2.26-fold decrease in OFC, and this decrease was significant when compared to IR and TQ alone. IR alone in patient 2 significantly decreased the OFC by 1.49-fold, whereas combination of 5 µM TQ and IR reduced OFC by 1.68-fold (Figure 7d). Combining 5 µM with IR reduced the size of organoids by more than 1-fold. In patient 3 (Figure 8d), IR alone significantly reduced the total count of organoids by more than 5-fold. The reduction in combination-treated organoids was comparable to the reduction by IR alone. Combining 5 µM TQ and IR reduced the size of organoids by more than 1-fold and this reduction was significant when compared to TQ alone.

Using immunofluorescence, we characterized the organoids established from the three patients for CRC stem cell markers. Positive staining of CD44 and CK19 demonstrated the presence of stem-like cells within the bulk of PDOs (Figure 6c, Figure 7c and Figure 8c). Immunofluorescent staining of the parental tumor tissues with CK19 showed a positive expression, in consistency with the established organoids.

The three patients were all treatment-naïve but with different clinical manifestations (Table S5). Patient 1 had rectal mucinous adenocarcinoma (pT2 stage). Patients 2 and 3 had moderately differentiated (grade 2) pT2 and pT3 sigmoid colon adenocarcinoma, respectively.

## 4. Discussion

This study was designed to investigate the radiosensitizing effect of TQ and its underlying mechanisms of action in different CRC cells grown in 2D and 3D cultures, and in PDOs. Our study is the first to show a radiosensitizing potential of TQ in colorectal CSCs and in PDOs. In 3D colonosphere cultures, radiosensitization by TQ correlated with significant inhibition of DNA repair and reduction in the expression of CRC stem cell markers, CD44 and CK19, which confirms the efficacy of combination treatment in eradicating CSCs. In addition, the combination treatment downregulated the expression of CD133, β catenin and NF-κB, molecules involved in stemness and radiation therapy, while inducing p53 and p21 expression. In our 2D model, combination of TQ and IR inhibited cell proliferation, viability, and colony survival. It also induced G2/M arrest, which was associated with DNA damage and persistent expression of γH2AX. Mechanistically, TQ and IR combination upregulated p53 and p21, and targeted NF-κB, MEK/ERK, Wnt/β catenin, and p-mTOR pathways involved in radioresistance.

Colorectal cancer remains among the most lethal and prevalent malignancies worldwide. Treatments for CRC include surgical resection, radiotherapy, ablative therapies for metastases, and palliative chemotherapy [34]. However, cancer recurrence may occur due to the resistance of CSCs to conventional therapies, including radiotherapy. For the first part of the study, we used a panel of CRC cells with different mutations and sensitivity to TQ and showed that TQ sensitized these cells to IR, independent of their p53 or K-ras status. IR alone had reversible inhibitory effects on the proliferation of all cell lines and no anti-proliferative effects on HT29 cells. Combining IR with TQ led to a dose-dependent reduction in proliferation of all CRC cells; however, effects were similar to TQ alone. Notably, MTT measures cellular metabolic activity as an indicator of cell proliferation, and thus may not be the ideal assay to clearly see the effect of combination treatments. On the other hand, trypan blue assay showed that combining TQ with IR reduced the viability of CRC cells more than TQ or IR alone. Clonogenic survival of irradiated HCT116 and HT29 cells was reduced by TQ, indicating radiosensitization through inhibition of long-term proliferation. This long-term inhibitory effect of TQ has been previously shown in irradiated breast cancer and HNSCC cell lines [23,35].

For subsequent experiments, HCT116 and HT29 cells were chosen to elucidate potential mechanisms of sensitization by TQ. Interestingly, combining TQ with IR enhanced arrest at the G2/M phase, during which cells are most vulnerable to irradiation, consistent with the mechanism of action of potent radiosensitizing agents studied in the context of CRC [36,37,38,39]. ATM and ATR are involved in the regulation of G2/M checkpoint. They also play important roles in the cellular response to DNA damage [40]. Analysis of ATM and ATR showed an upregulation of their active forms at early times following irradiation in HCT116 and HT29 cells treated with TQ and IR. However, only p-ATR upregulation lasted for a longer period and significant high levels were maintained 24 h post irradiation in combination-treated cells. Double strand breaks (DSBs) activate ATM and ATR, which in turn phosphorylate and activate chk2 and chk1, respectively [41]. ATM is the major kinase that phosphorylates H2AX, but other kinases can substitute for ATM, including ATR and DNA-PKc [42,43]. Phosphorylation of H2AX leads to recruitment of DNA repair machinery. Importantly, during DSB resection, an ATM-to-ATR switch is observed, which results in activation of chk1 and G2/M phase arrest [41,44]. These observations could explain the persistent high levels of p-ATR but not p-ATM 24 h post irradiation, suggesting that DSBs induced by IR result in ATM and ATR activation at first and are thereafter dependent on ATR activation and cell cycle arrest to repair DNA damage.

We next examined the extent of DNA damage in treated cells by analyzing the expression of γH2AX. We [28] and others [45,46,47,48] have used γH2AX foci as a marker for TQ- and IR-induced DNA damage. Our results showed highest number of γH2AX foci 10 min post IR, suggesting that the repair of damage began early in CRC cells. Interestingly, we found a persistent upregulation of this DNA damage marker upon treatment with TQ prior to irradiation. This suggests that TQ sensitizes HCT116 and HT29 cells to radiation through maintaining constitutive phosphorylation of H2AX, indicating a delayed repair of radiation-induced DSB, resulting in cell cycle arrest and possibly apoptosis.

To understand what molecular pathways could be targeted by TQ and IR, we focused on pathways implicated in radiation therapy. A recent report has documented frequent activation of mTOR in CRC liver metastasis [49]. Importantly, irradiation induces mTOR phosphorylation and activation, which contributes to cancer metastasis [50,51]. Our results showed that TQ alone and in combination with IR was successful in reversing the induction of p-mTOR in control and irradiated cells. It has been postulated that targeting Wnt and MEK/ERK pathways may be of clinical significance for patients with metastatic CRC [52]. Moreover, MEK/ERK pathway is known to play a role in cell survival after radiation [53]. Therefore, we were interested in studying the potential effect of combination treatment on these pathways. According to our findings, TQ and IR combination reduced the expression of β catenin and MEK, suggesting that the combination treatment inhibits survival and metastasis of CRC cells possibly through targeting these pathways. Interestingly, TQ and IR reduced the expression of NF-κB, which is involved in radioresistance. A previous study showed that TQ sensitized CRC cells to cisplatin by inhibiting activation of NF-κB [14]. Combination treatment induced the upregulation of p53 and p21 in HCT116 cells, and this induction was comparable to that of IR treatment and was associated with G2/M arrest. The induction of cell cycle arrest by p53 and p21 responses following DNA damage is well studied [54]. Combination treatment induced p53 but not p21 in HT29 cells. Treating HCT116 and HT29 with TQ and IR reduced the expression of the stem cell marker CD133 in HT29 but not in HCT116 cells, suggesting that combination treatment may be targeting different CRC stem cell markers in these cells. TQ was shown to target other molecular pathways, resulting in sensitization to radiation [21,22]. TQ alone or in combination with paclitaxel enhanced radiosensitivity of MCF7 and MDA-MB-231 breast cancer cells by inhibiting radiation-induced colony formation, migration and invasion, and epithelial-mesenchymal transition (EMT) via restoring E-cadherin and decreasing TGF-β, integrin αV, MMP9, and MMP2 expression in irradiated cells [22]. Combining TQ with radiation enhanced its anti-proliferative effects and reduced the colony forming ability of MCF7 and T47D as compared to individual treatments and this was associated with enhanced apoptosis and changes in cell cycle regulation [21]. TQ also demonstrated synergistic effects when combined with radiation in HNSCC through inhibition of proliferation [23] and enhanced the effect of gamma knife on apoptosis and DNA damage in B16-F10 melanoma cells by modulating the JAK2/STAT3 pathway [24].

Given that CSCs are the population responsible for the resistance to radiation, we sought to study the radiosensitizing potential of TQ in CRC stem/progenitor cells enriched from HCT116 and HT29 cell lines. TQ alone inhibited the self-renewal capacity of spheres derived from both cell lines, which is consistent with recent findings from our lab [28]. The radiosensitizing effect of TQ was observed in colonospheres at G1 and led to depletion of CSCs at G5, which suggests that combination of TQ and IR effectively targets CSCs and exerts a stronger inhibitory effect than either treatment alone. Studies investigating the effect of TQ on CSCs are very few. Recent studies have shown that combining TQ with chemotherapeutic agents or natural compounds enhances inhibition of CSCs [55,56,57]. The combined treatment of TQ and emodin enhanced eradication of CD44+/CD24− CSCS population, when compared to either treatment alone. Further analysis showed inhibition of stemness through downregulation of CSC markers, OCT-4 and SOX-2 [57]. Combining TQ with 5-FU depleted CD133+ cancer stem cells and reduced self-renewal potential of CRC stem/progenitor cells, possibly through downregulation of Wnt and PI3K pathways involved in stemness [20].

To understand the mechanism of sensitization by TQ in 3D colonospheres, we determined the effect of combination treatment on DNA damage and on the expression of several CRC stem cell markers and major stem cell regulatory pathways. Our results demonstrated that TQ or IR alone did not induce significant DNA damage, as shown by γH2AX data, which translates into a highly efficient activation of DDR and repair in CSCs [58]. Interestingly, combination of TQ and IR led to increased phosphorylation of H2AX, suggesting diminished DNA repair ability, which results in radiosensitization of the CSCs. Moreover, we showed that CD44 was highly expressed in control and irradiated spheres and that TQ sensitized HCT116 and HT29 spheres by reducing its expression. The inhibition of sphere forming ability seen here correlated with the decrease in this stem cell marker upon treatment with TQ alone or TQ and IR combination. While combination treatment had no effect on CK8 and CK19 expression in CSCs enriched from HT29 cells, it led to a reduction in the expression of these markers in HCT116 cells. This suggests a different mechanism of stemness inhibition by TQ and IR in different CSCs. Importantly, TQ and IR combination also reduced the expression of CD133 in HCT116 and HT29 spheres, which is associated with migration and stemness in CRC [59]. CD44 and CD133 are recognized among others as putative CRC stem cell markers [60,61]. The high expression of CD44 and CD133 in CSCs that were derived from HCT116 and HT29 cells confirms enrichment of CSCs in our 3D cultures of spheroids. CD44 is a surface glycoprotein that plays a role in several key processes including survival, stemness, and cell migration. It is important to note that it is partly activated by Wnt/β catenin pathway [62], which explains the decrease in its expression, in line with the observed downregulation of β catenin in HCT116 and HT29 spheres treated with TQ and IR. As for CK19, which was significantly downregulated in HCT116 spheres treated with combination, it is known to be a marker of CSCs and is used to identify circulating CRC stem cells and to confirm their epithelial nature [63]. Similar to 2D cultures, combination treatment decreased the expression of NF-κB, and increased the expression of p53 and p21.

Different responses to individual and combination treatments were observed in PDOs which could be explained by the differences in their clinical and histopathological characteristics, including tumor location and stage. Organoids derived from patient 1 were the most resistant to IR. Notably, combining TQ with IR had a significant effect on these organoids by reducing their total count and size. Interestingly, the response of HT29 organoids to combination treatment was similar to organoids derived from patient 1. In patient 2, IR alone was good as a standalone treatment, and had similar effects to combination treatment on the organoid forming ability. However, organoid size was reduced only in combination-treated organoids, suggesting inhibition of proliferation in response to TQ and IR. Patient 3 was the most sensitive to irradiation, and combination of TQ with IR resulted in similar inhibition by IR alone on OFC. Interestingly, TQ was good as a standalone treatment for PDOs derived from patient 3. Similar to patient 2, only combination treatment had an inhibitory effect on organoid size.

CD44 has prognostic and clinical value in CRC and is being used to predict poor prognosis and metastasis [64]. High expression of CD44 has been associated with self-renewal, tumor initiation, metastasis, and resistance to apoptosis, chemotherapy, and radiotherapy [65,66]. CK19 has been also shown to be associated with colon carcinogenesis [67]. Therefore, the high expression of these markers in the established organoids, as well as in the corresponding tumor tissues, confirms the presence of stem cells and the maintenance of tumor tissue characteristics in these 3D models.

The present study has several limitations. We acknowledge that the patient sample size is small and dependent on the availability of tissues at the time of the study. Additionally, among the few CRC samples we received, only three successfully formed organoids in culture, possibly due to limitations in tissue quality and size, which hindered organoid derivation. In addition, PDO staining was limited by the low number of PDOs generated, mainly in treated groups. It remains essential to evaluate the radiosensitizing potential of TQ on additional patient samples and to link the differential responses to clinical data. Moreover, future studies should determine the radiosensitizing effects of TQ on more CRC cell lines and the mechanisms of radiosensitization in 2D and 3D cultures. It would be interesting to study the effect on several pathways and molecules implicated in radioresistance and survival (PI3K/Akt/mTOR, MEK/ERK, and Notch) and DNA damage repair (DNA-PKc, RAD51, BRCA and others). Mechanistic studies in vitro could be followed by validation in PDOs, which have more clinical relevance relative to cell lines and offer a suitable tool for predicting clinical response and implementing personalized medicine. Based on future experiments, our data hold promise for the potential use of TQ as a radiosensitizer, given its minimal toxicity to normal tissues and its well-established anti-cancer activities.

## 5. Conclusions

In this study, we reported for the first time a radiosensitizing potential of TQ in 2D and 3D cultures of CRC cells and in PDOs. Importantly, radiosensitization was associated with inhibition of cell viability, long-term survival, and DNA repair. TQ in combination with radiation also targeted pathways implicated in radiation therapy and self-renewal capacity in cancer cells. Interestingly, PDOs (patient 1) that were less responsive to radiation alone had a significant response to combination of TQ and radiation. Based on these results, combination of TQ with radiation might represent a useful tool for targeting cancer cells and stem/progenitor cells.

## Data Availability

Data is contained within the article.

## Supplementary Materials

The following are available online at https://www.mdpi.com/article/10.3390/cancers14061363/s1, Figure S1: TQ and radiation combination is non-toxic to non-tumorigenic intestinal cells. Figure S2: Effect of TQ and IR on CRC cell proliferation. Figure S3: TQ sensitizes colorectal cancer cells to radiation that leads to reduction in their cell viability and colony formation ability. Figure S4: TQ enhances radiation-induced cell cycle arrest at G2/M phase in colorectal cancer cells. Figure S5: TQ sensitizes colorectal cancer cells to radiation through targeting major pathways implicated in radiation therapy. Figure S6: TQ enhances the effect of radiation on self-renewal capacity of colorectal cancer stem/progenitor cells. Figure S7: TQ radiosensitization of colorectal cancer stem/progenitor cells leads to inhibition of stemness. Figure S8: TQ radiosensitizes colorectal cancer cell line-derived organoids and reduces their organoid-forming ability. Figure S9: Whole Western blot membranes for HCT116 (2D). Figure S10: Whole Western blot membranes for HT29 (2D). Figure S11: Whole Western blot membranes for HCT116 (3D). Figure S12: Whole Western blot membranes for HT29 (3D). Table S1: List of primary and secondary antibodies used in immunofluorescent staining. Table S2: List of primary and secondary antibodies used in western blot experiments. Table S3: Significance between different groups stained for p-ATM, p-ATR, and γ H2AX in HCT116 cells irradiated for 0 min, 10 min, or 24 h. Table S4: Significance between different groups stained for p-ATM, p-ATR, and γ H2AX in HT29 cells irradiated for 0 min, 10 min, or 24 h. Table S5: Colorectal cancer patients’ clinical and histopathologic characteristics.
