# Correction: Al Bitar et al. Thymoquinone Radiosensitizes Human Colorectal Cancer Cells in 2D and 3D Culture Models. *Cancers* 2022, *14*, 1363

**DOI:** 10.3390/cancers17152449

**Published:** 2025-07-24

**Authors:** Samar Al Bitar, Farah Ballout, Alissar Monzer, Mariam Kanso, Nour Saheb, Deborah Mukherji, Walid Faraj, Ayman Tawil, Samer Doughan, Maher Hussein, Wassim Abou-Kheir, Hala Gali-Muhtasib

**Affiliations:** 1 Department of Biology, American University of Beirut, Beirut 1107-2020, Lebanon; sfa28@mail.aub.edu (S.A.B.); frb03@mail.aub.edu (F.B.); aam48@mail.aub.edu (A.M.); 2 Division of General Surgery, Department of Surgery, American University of Beirut Medical Center, Beirut 1107-2020, Lebanon; mk212@aub.edu.lb (M.K.); wf07@aub.edu.lb (W.F.); sd65@aub.edu.lb (S.D.); mh45@aub.edu.lb (M.H.); 3 Department of Pathology and Laboratory Medicine, American University of Beirut Medical Center, Beirut 1107-2020, Lebanon; ns141@aub.edu.lb (N.S.); at04@aub.edu.lb (A.T.); 4 Division of Hematology/Oncology, Department of Internal Medicine, Faculty of Medicine, American University of Beirut Medical Center, Beirut 1107-2020, Lebanon; dm25@aub.edu.lb; 5 Department of Anatomy, Cell Biology and Physiological Sciences, Faculty of Medicine, American University of Beirut, Beirut 1107-2020, Lebanon

## Error in Figure 7

In the original publication [1], there was a mistake in Figure 7d as published. The image labeled TQ 5µM (5x) is wrong and mistakenly used, as it is the same as the one labeled TQ 3µM. The corrected image for TQ 5µM (5x) appears below. The authors state that the scientific conclusions are unaffected. This correction was approved by the Academic Editor. The original publication has also been updated.


