# Supplementary material for: Thymoquinone Radiosensitizes Human Colorectal Cancer Cells in 2D and 3D Culture Models
Source: Cancers (Basel). 2022 Mar 8;14(6):1363. doi: 10.3390/cancers14061363 (PMC8945905; doi:10.3390/cancers14061363)
Supplement: Supplementary file 1 [file cancers-14-01363-s001.zip › cancers-1555651 supplementary.pdf]

Supplementary Materials

Thymoquinone Radiosensitizes Human Colorectal Cancer Cells in 2D and 3D Culture Models

Samar Al Bitar, Farah Ballout, Alissar Monzer, Mariam Kanso, Nour Saheb, Deborah Mukherji, Walid Faraj, Ayman Tawil, Samer Doughan, Maher Hussein, Wassim Abou-Kheir and Hala Gali-Muhtasib

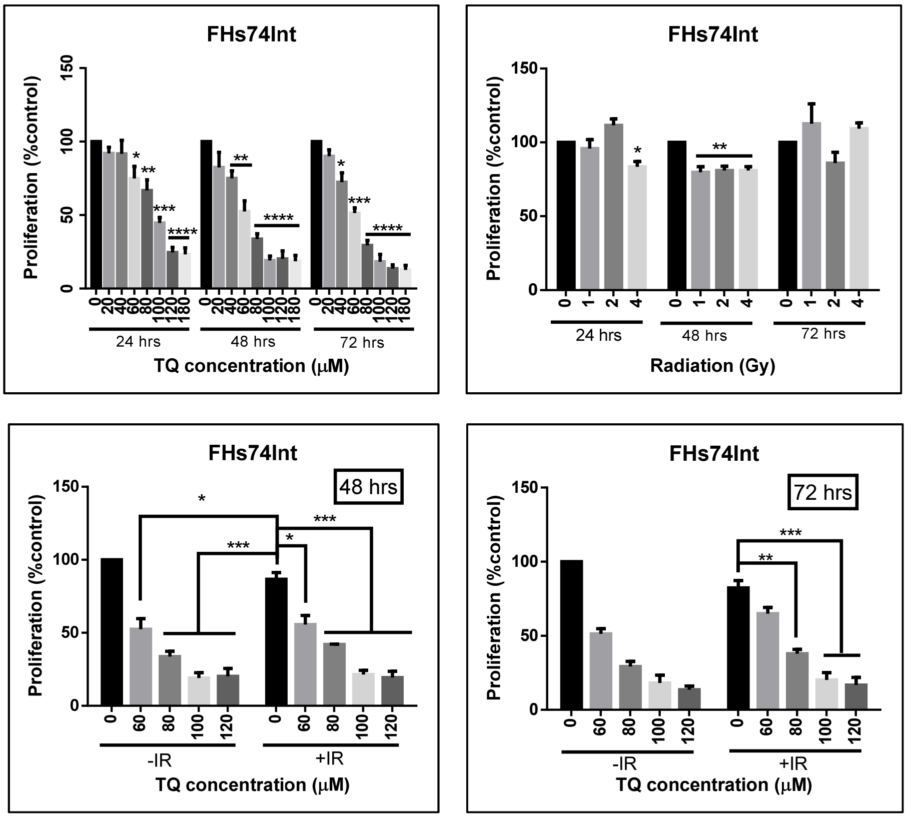

Figure S1. TQ and radiation combination is non-toxic to non-tumorigenic intestinal cells. FHs74Int cells were incubated with or without TQ, or with or without radiation for 24, 48, or 72 h or were treated with TQ followed by irradiation at a dose of 2 Gy for 48 and 72 h (combination). At the specific time point, cell proliferation was determined using MTT assay. Results are expressed as percentage of the studied group as compared to its control. Data represent an average of three independent experiments. The data are reported as mean ± SEM (* $p < 0.05$; ** $p < 0.01$; *** $p < 0.001$, **** $p < 0.0001$).

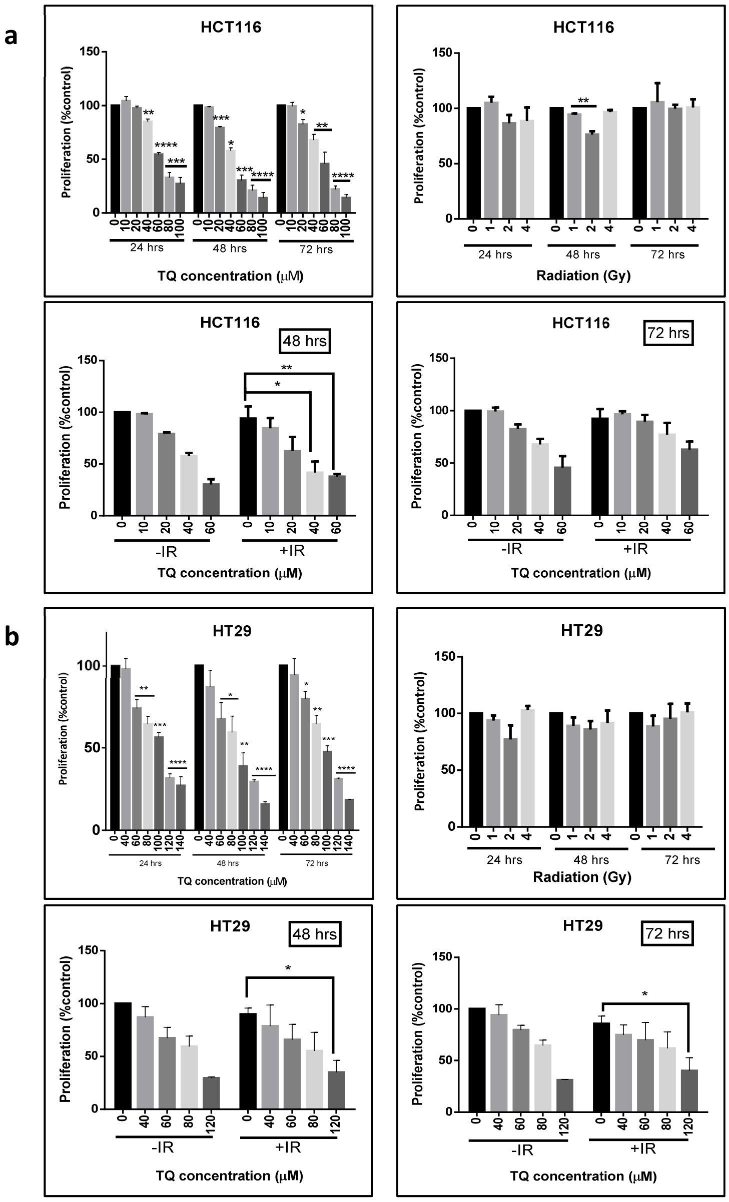

Figure S2. Effects of TQ and IR on CRC cell proliferation. HCT116 (**a**) and HT29 (**b**) cells were either left untreated or were incubated with TQ alone, IR alone (2 Gy) or combinations for 24, 48, or 72 hrs. At the specific time point, cell proliferation was determined using MTT. Results are expressed as percentage of the studied group as compared to its control. Data represent an average of three independent experiments. The data are reported as mean ± SEM (* $p < 0.05$; ** $p < 0.01$; *** $p < 0.001$, **** $p < 0.0001$).

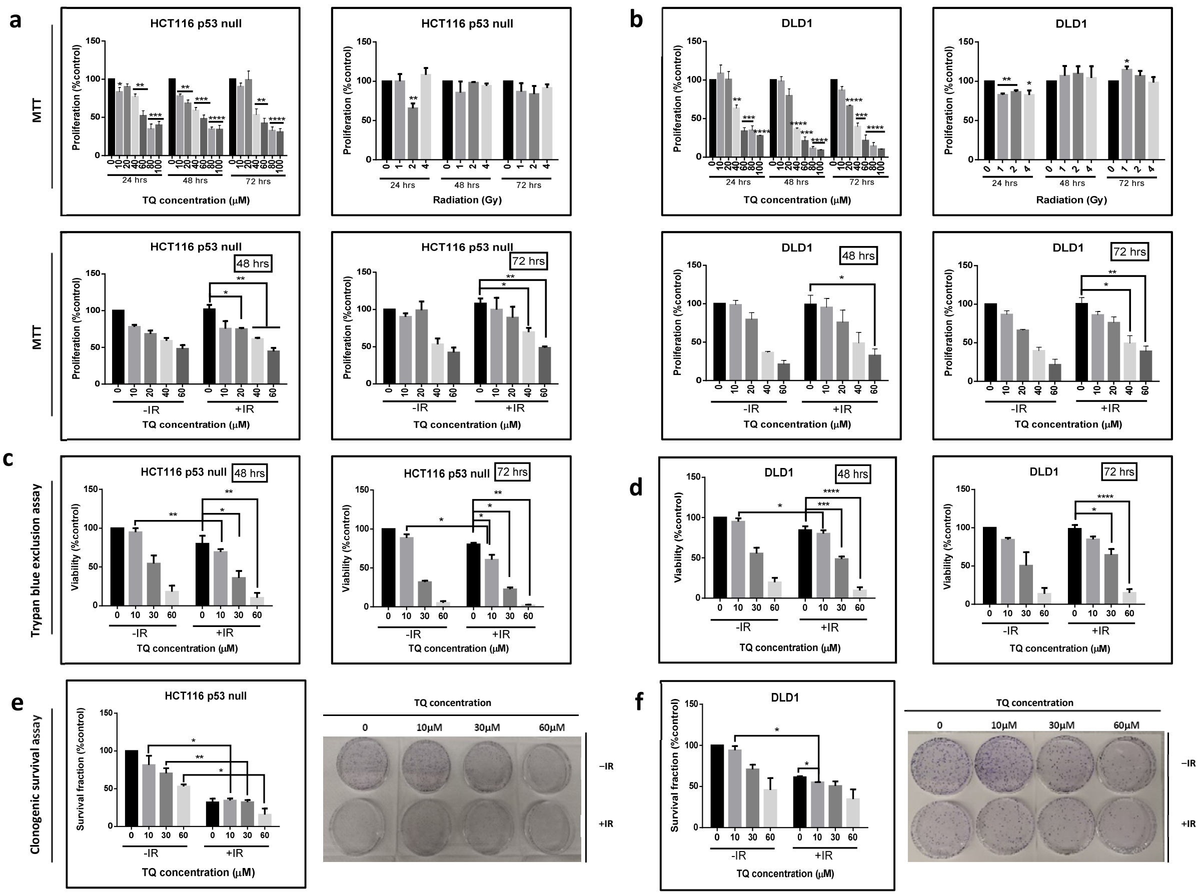

Figure S3. TQ sensitizes colorectal cancer cells to radiation that leads to reduction in their cell viability and colony formation ability. (**a**–**d**) HCT116 p53 null and DLD1 colorectal cancer cells were either left untreated or were incubated with TQ alone, IR alone (2 Gy) or combinations for 24, 48, or 72 h. At the specific time point, cell proliferation and viability were determined using MTT (**a**,**b**) and trypan blue exclusion assay (**c**,**d**), respectively. (**e**,**f**) Colony formation assay was used to determine effect of TQ and IR on the long-term survival of colorectal cancer cells. Cells were treated with TQ, IR, or TQ + IR, after which they were collected and seeded in treatment-free media at low density. After 7–10 days, the resulting colonies were fixed, stained with crystal violet and counted. Results are expressed as percentage of the studied group as compared to its control. Data represent an average of three independent experiments. The data are reported as mean ± SEM (* $p < 0.05$; ** $p < 0.01$; *** $p < 0.001$, **** $p < 0.0001$).

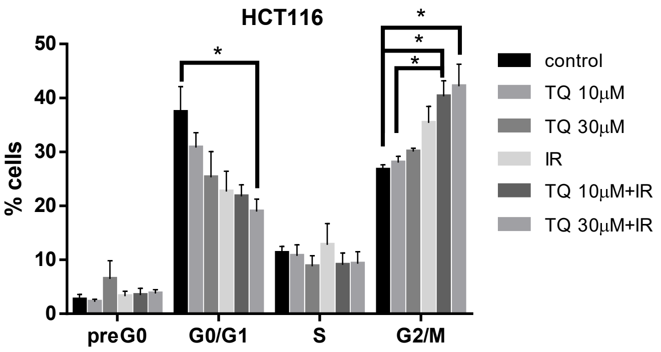

(**a**)

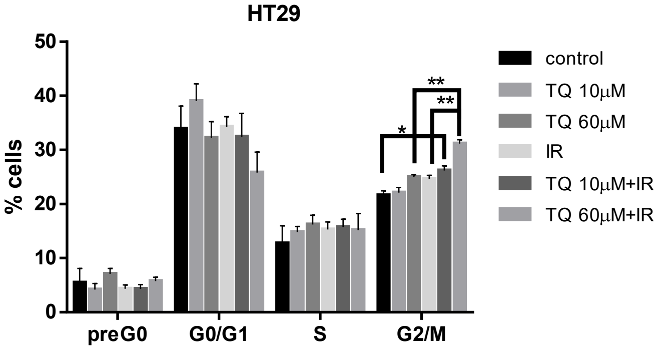

(**b**)

Figure S4. TQ enhances radiation-induced cell cycle arrest at G2/M phase in colorectal cancer cells. Cell cycle was evaluated by flow cytometry using propidium iodide staining of HCT116 (**a**) and HT29 (**b**) cells after each treatment (control, TQ, IR, and TQ + IR) at 48 h. Data represent an average of three independent experiments and are reported as mean ± SEM (* $p < 0.05$; ** $p < 0.01$).

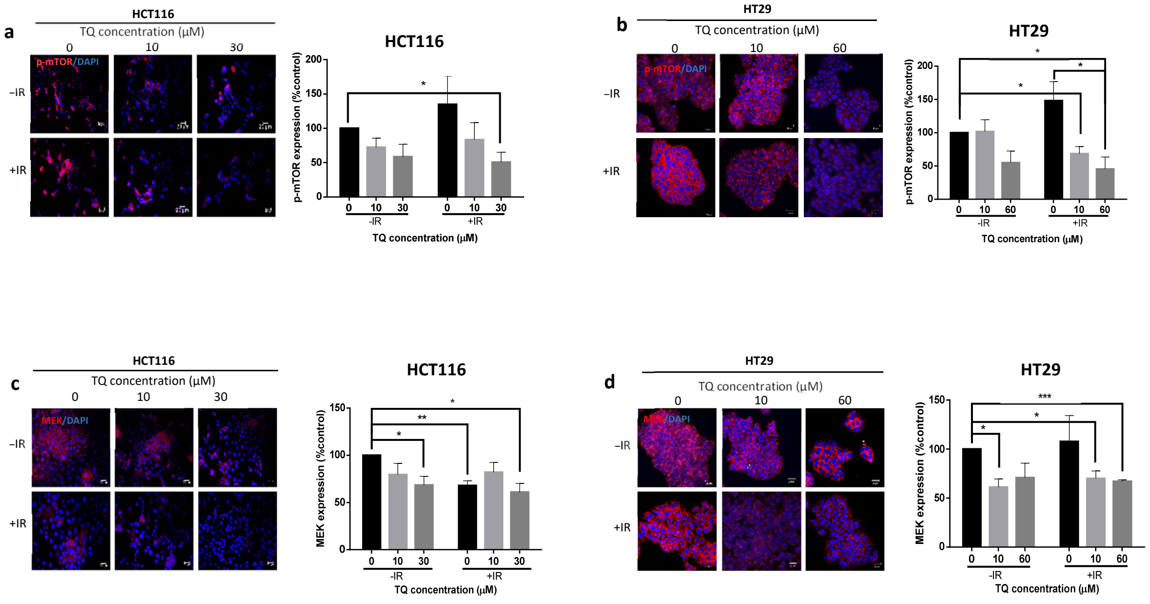

Figure S5. TQ sensitizes colorectal cancer cells to radiation through targeting major pathways implicated in radiation therapy. HCT116 and HT29 cells untreated and treated with TQ, IR, and combination of TQ and IR (TQ + IR) were immunofluorescently stained for p-mTOR (**a**,**b**) and MEK (**c**,**d**). Quantification of intensity was performed using Carl Zeiss Zen 2012 image software. Data represent an average of three independent experiments and are reported as mean ± SEM (* $p < 0.05$; ** $p < 0.01$; *** $p < 0.001$). Scale bar for immunofluorescent images is 20 μm.

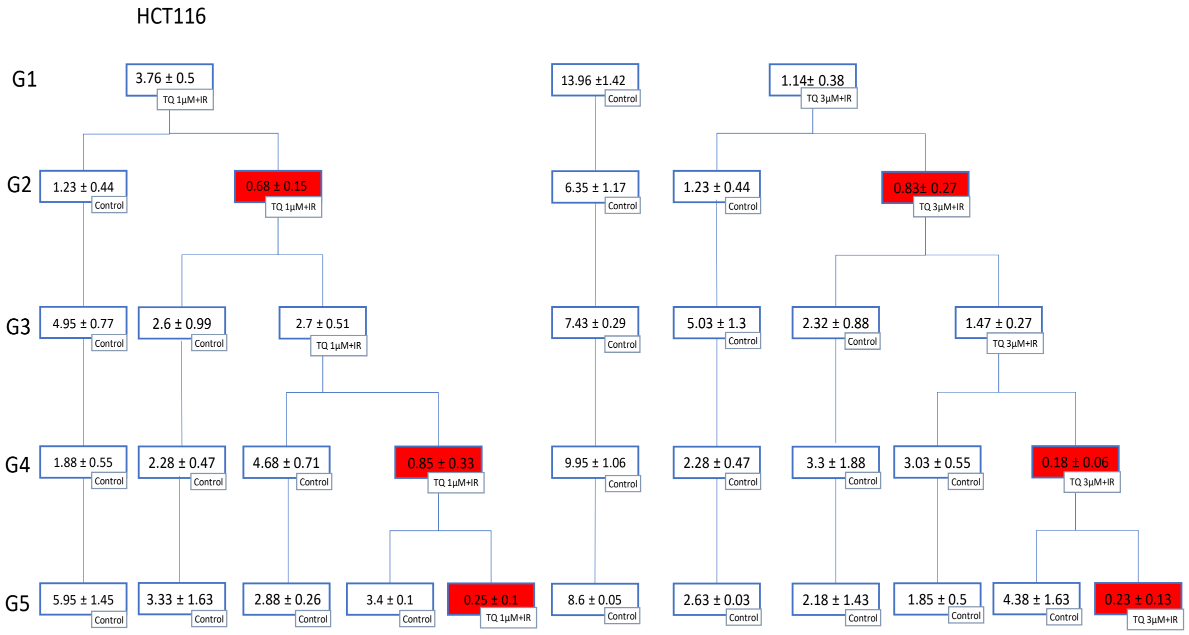

(a)

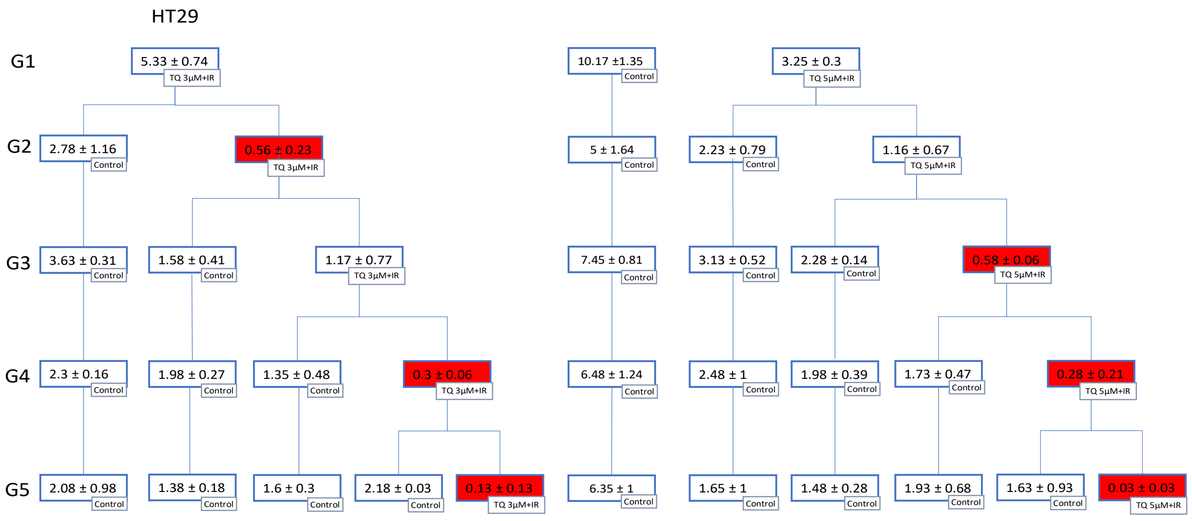

(b)

Figure S6. TQ enhances the effect of radiation on self-renewal capacity of colorectal cancer stem/progenitor cells. CRC stem cells were enriched from HCT116 (**a**) and HT29 (**b**) cell lines and treated with either TQ, IR, combinations, or media (control) (G1). After each propagation, cells that were initially treated

with combinations or media (control) were seeded into separate wells and cultured with or without treatment. Spheres were propagated for five generations in duplicates of each condition. SFU is counted and an average of three experiments is represented. The data are reported as mean ± SEM.

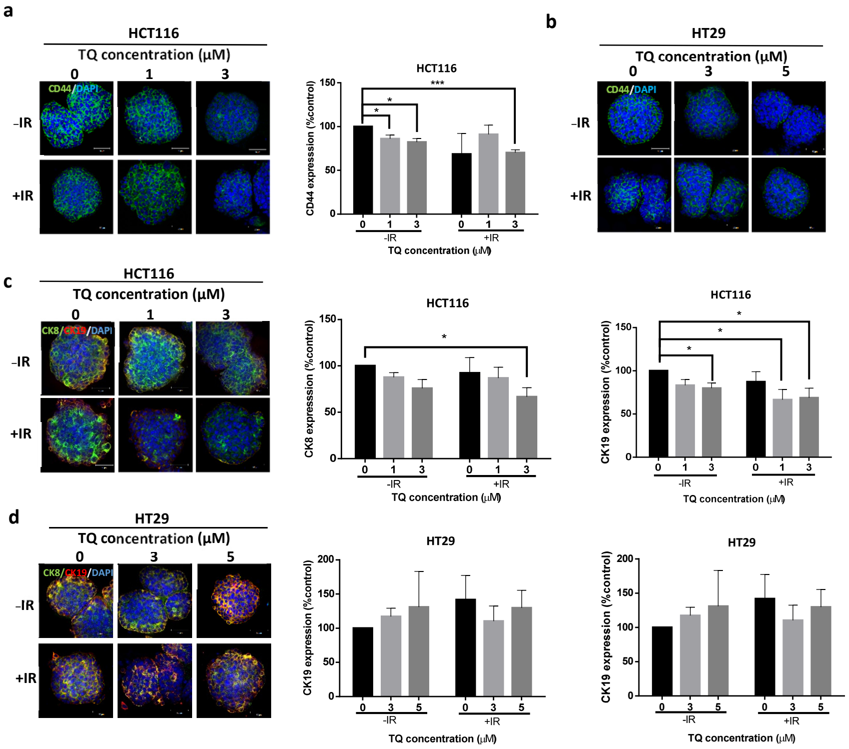

Figure S7. TQ radiosensitization of colorectal cancer stem/progenitor cells leads to inhibition of stemness. Representative images of TQ, IR, and TQ + IR treated HCT116 and HT29 G1 spheres after CD44 (**a**,**b**) and CK8/CK19 (**c**,**d**) staining. Intensity of stain in control, TQ, IR, and TQ + IR treated HCT116 and HT29 G1 spheres was performed using Carl Zeiss Zen 2012 image software. Stain intensity was normalized to size. Data represent an average of three independent experiments and are reported as mean ± SEM (* $p < 0.05$; ** $p < 0.01$; *** $p < 0.001$). Scale bar 20 μm.

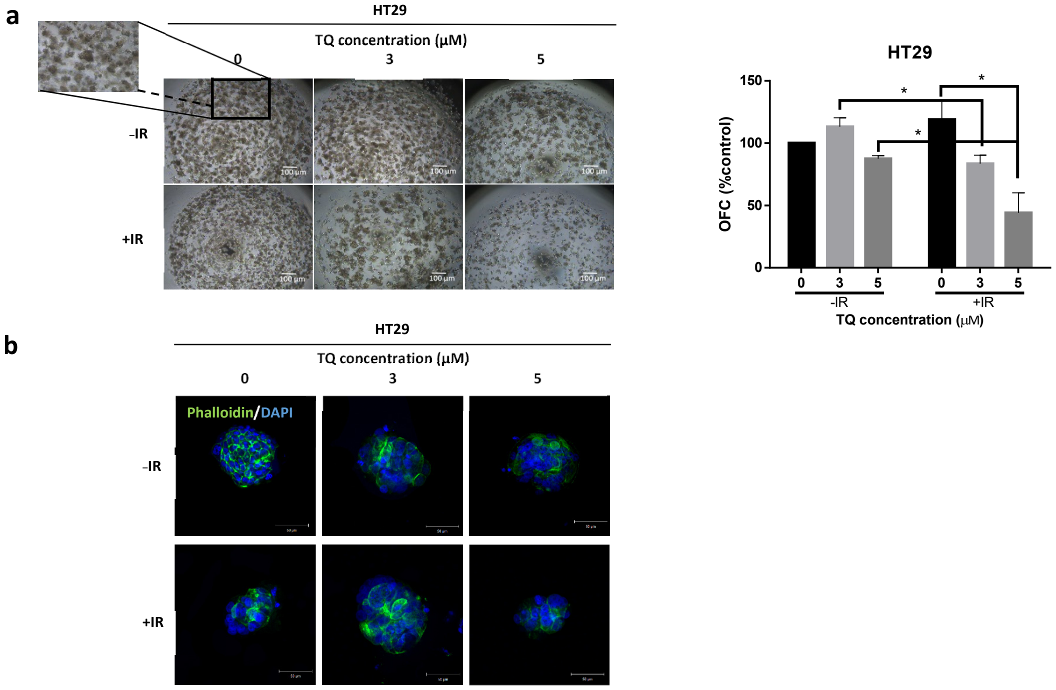

Figure S8. TQ radiosensitizes colorectal cancer cell line-derived organoids and reduces their organoid-forming ability. (**a**) Representative bright-field images of organoids derived from HT29 cells and treated with TQ (3 and 5 μM), radiation (2 Gy), or combinations. Images were visualized by Axiovert inverted microscope at 10× magnification and analyzed by Carl Zeiss Zen 2012 image software. Scale bar 100 μm. OFC is calculated and expressed as percentage of the treatment group as compared to its control according to the following formula: OFC (%control) = (number of organoids counted ÷ number of organoids in control group) ×100. HT29 cells were suspended in 90% Growth Factor reduced Matrigel and 10% serum-free colon media and allowed to grow in serum-free colon media (with or without treatment). Generated organoids are referred to as G1 organoids. Data represent an average of three independent experiments and are reported as mean ± SEM (* $p < 0.05$). (**b**) Representative immunofluorescence images of phalloidin-stained organoids were obtained using confocal microscopy. Scale bar 50 μm.

2D HCT116 p53

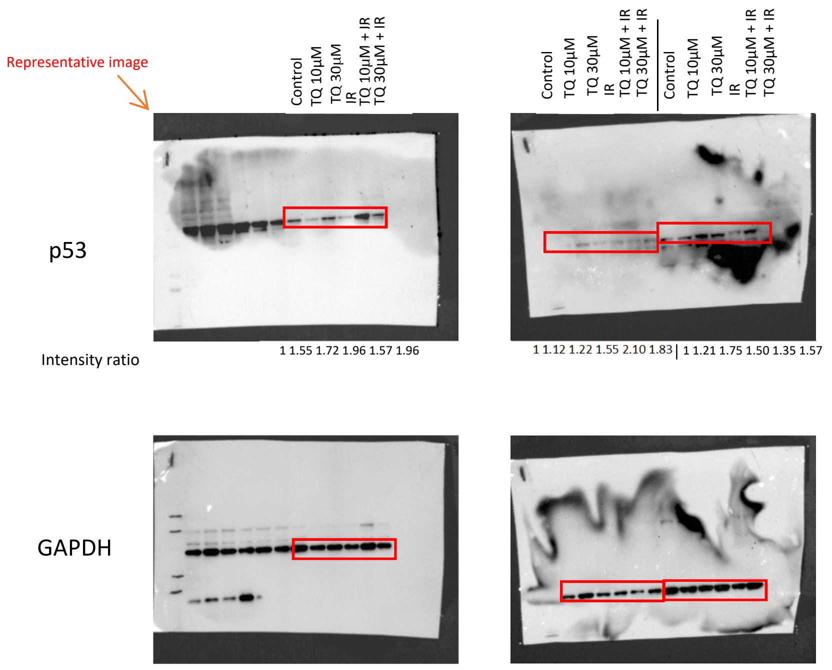

2D HCT116 p21

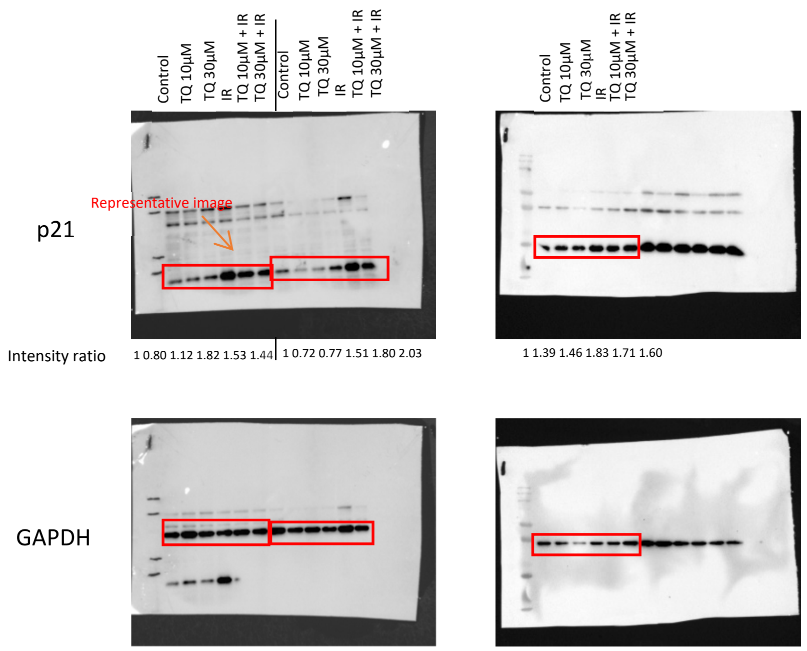

2D HCT116 NF-κB

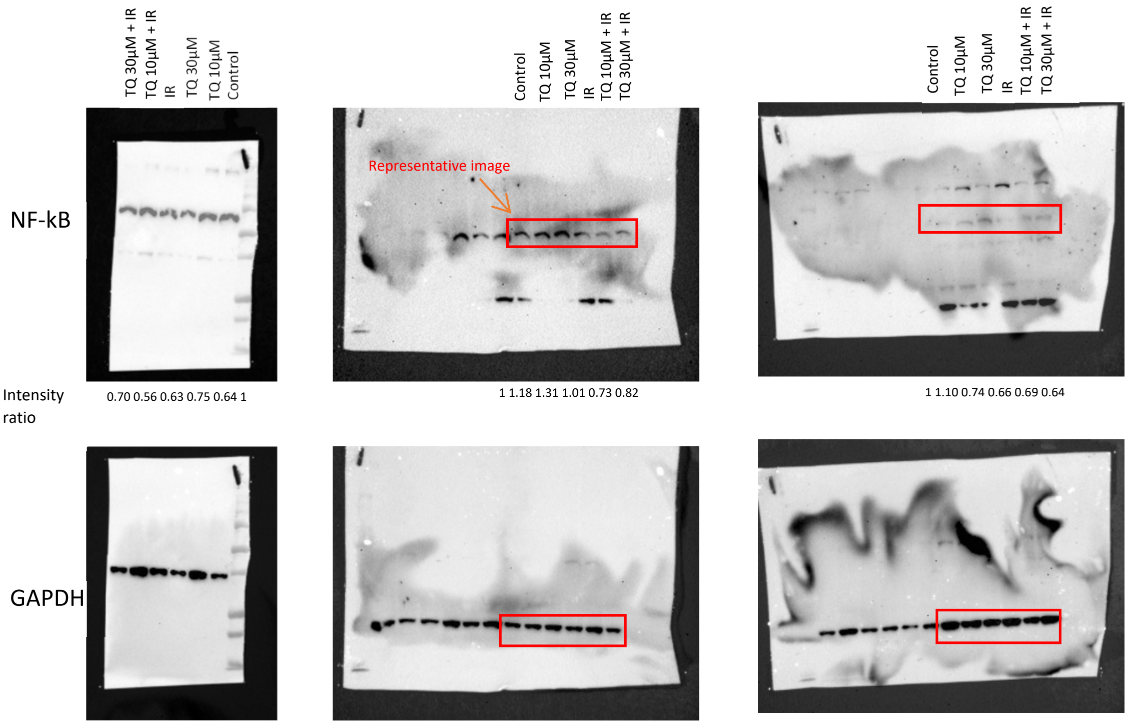

2D HCT116 β catenin

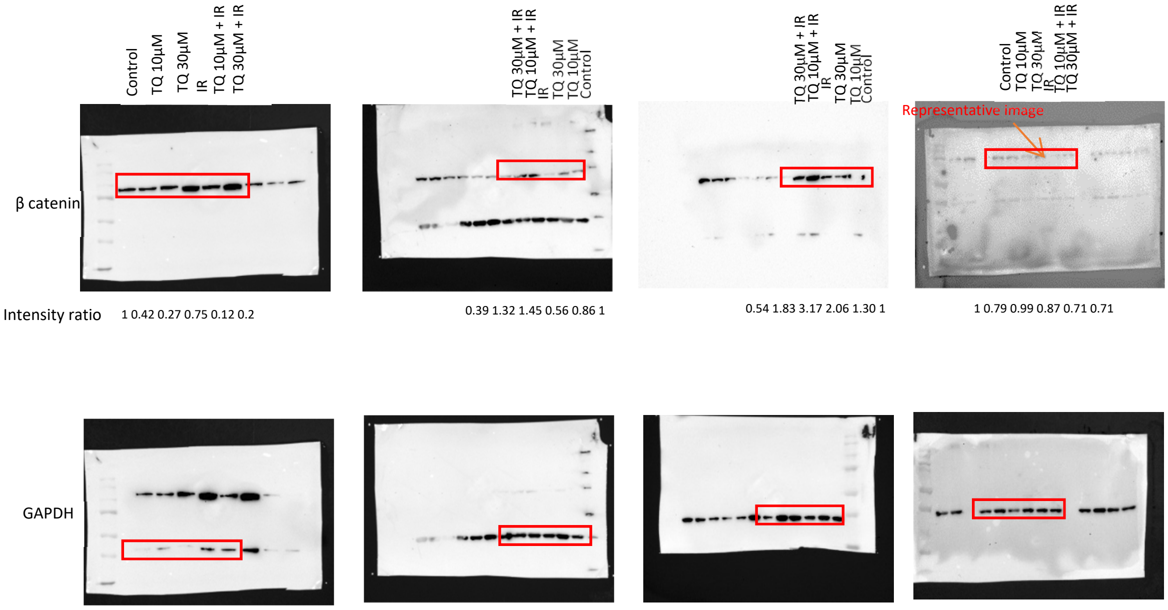

2D HCT116 CD133

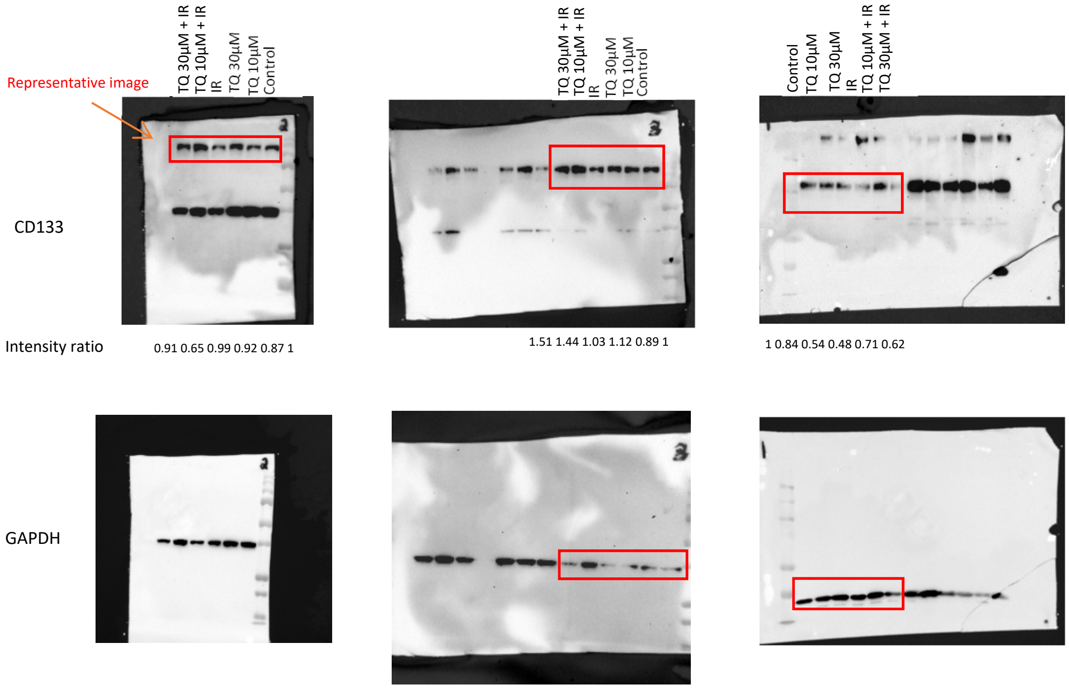

Figure S9. Whole western blot membranes for HCT116 (2D) (Figure 3a).

2D HT29 p53

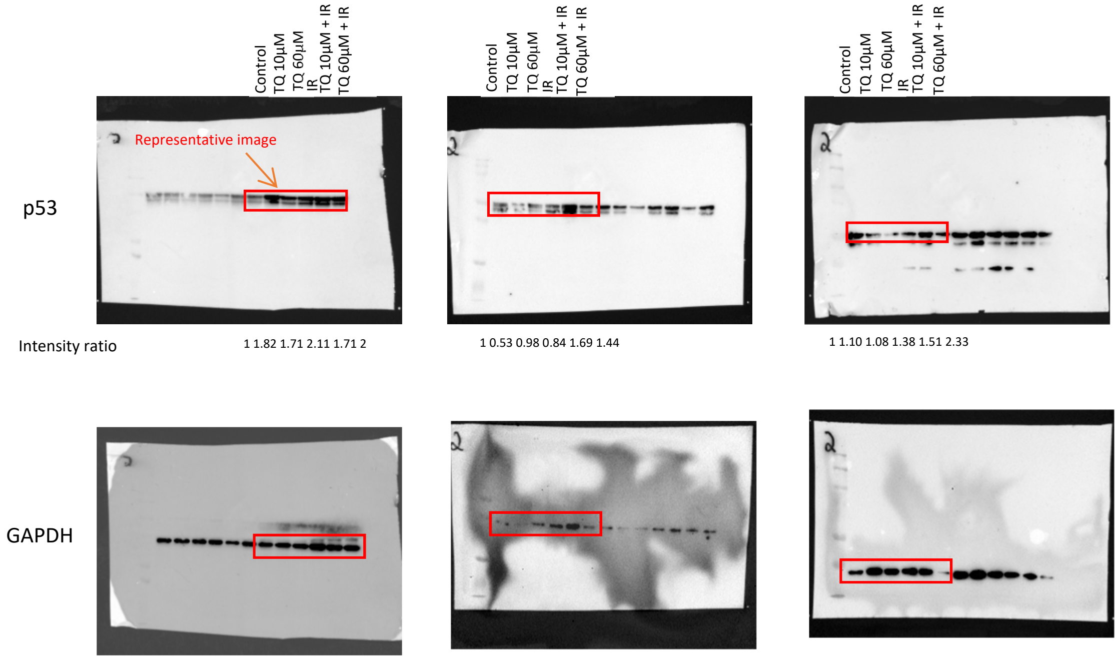

2D HT29 p21

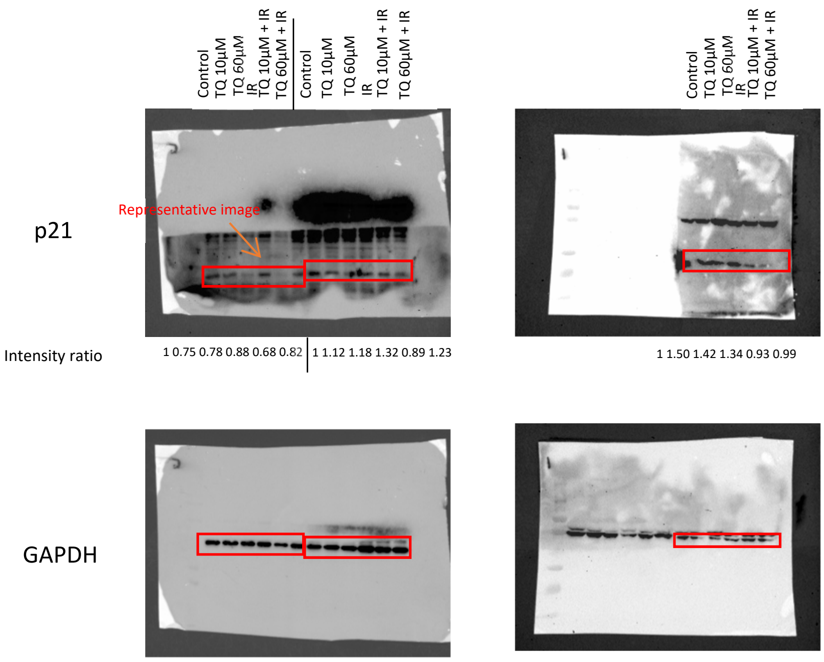

2D HT29 NF-κB

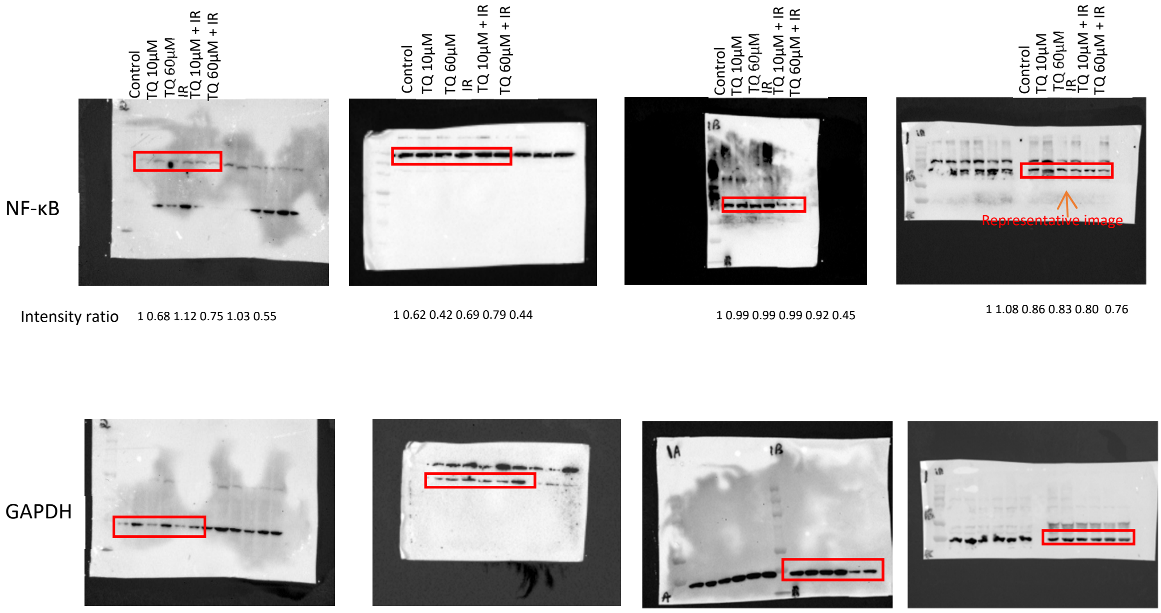

2D HT29 β catenin

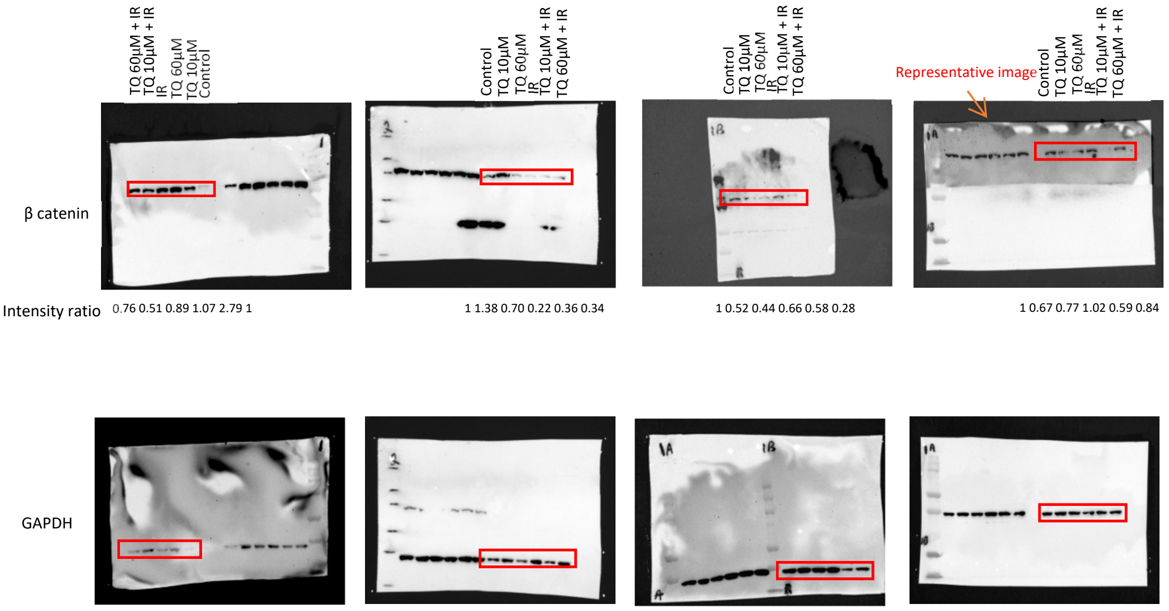

2D HT29 CD133

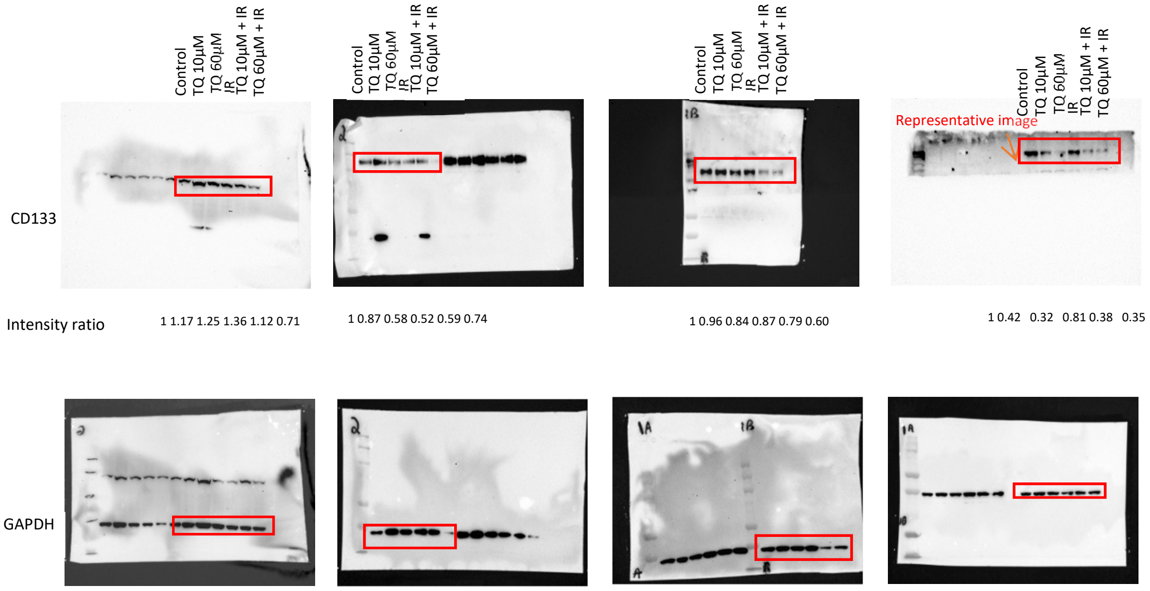

Figure S10. Whole western blot membranes for HT29 (2D) (Figure 3b).

3D HCT116 p53

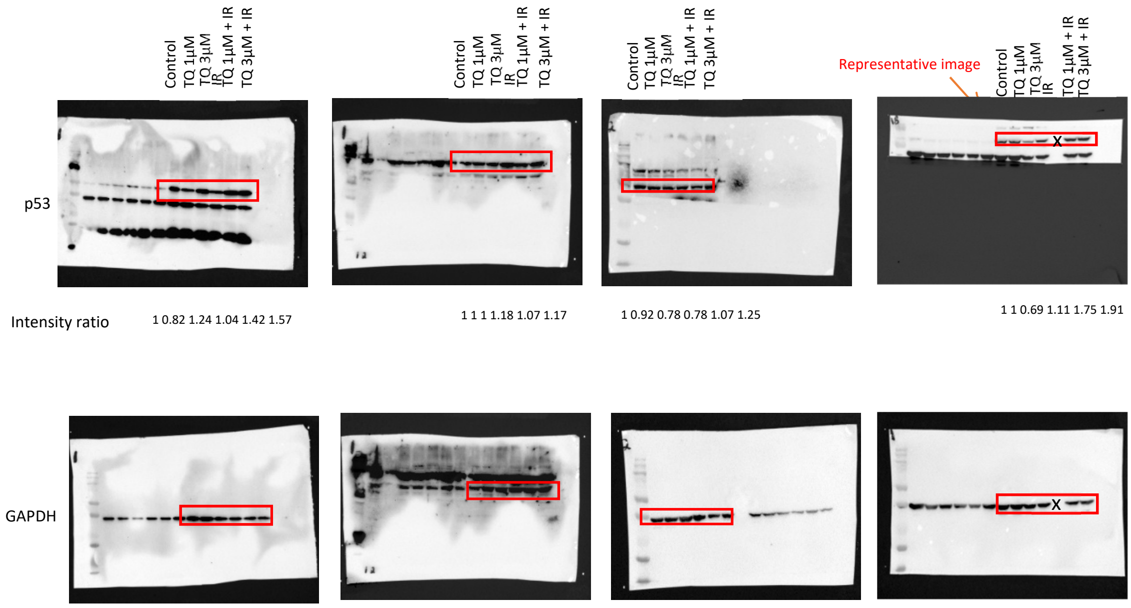

3D HCT116 p21

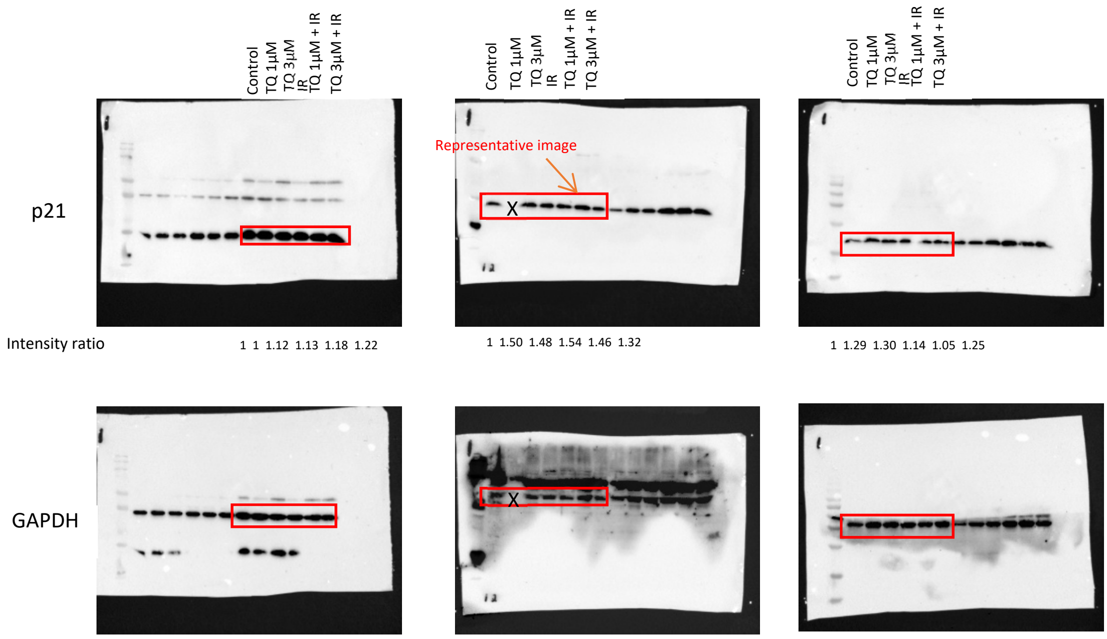

3D HCT116 NF-κB

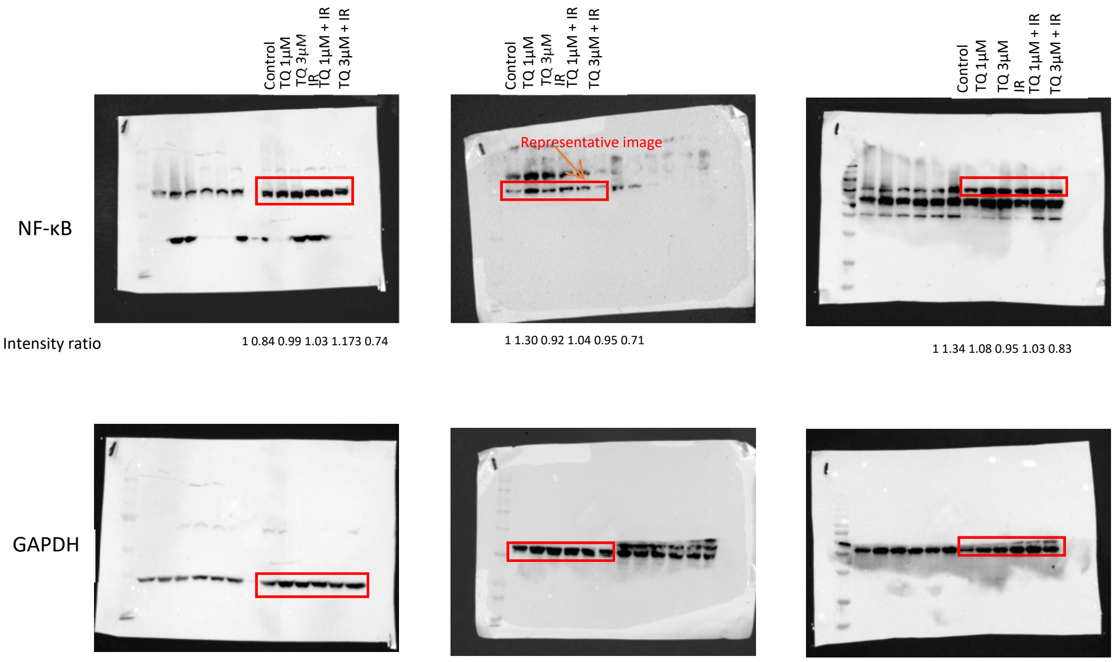

3D HCT116 β catenin

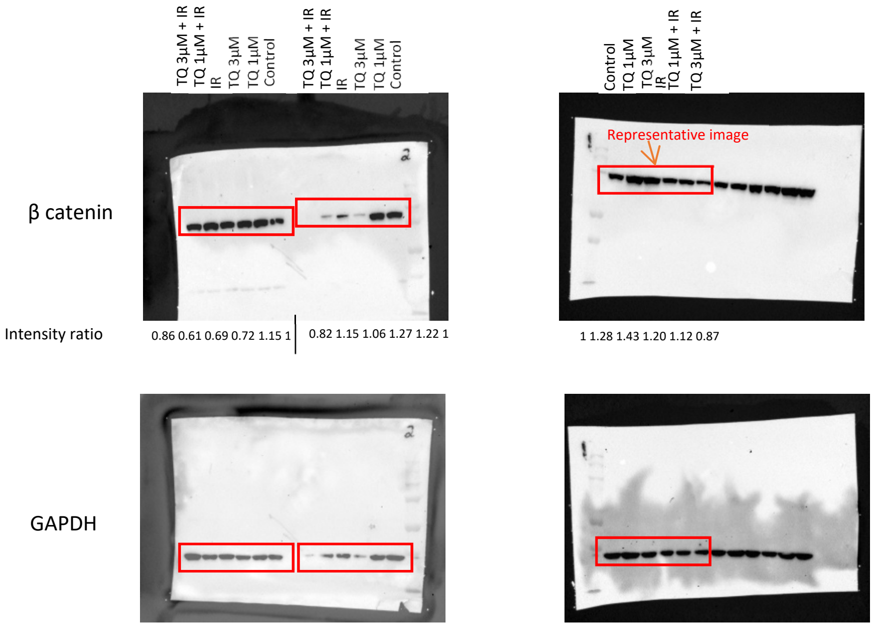

3D HCT116 CD133

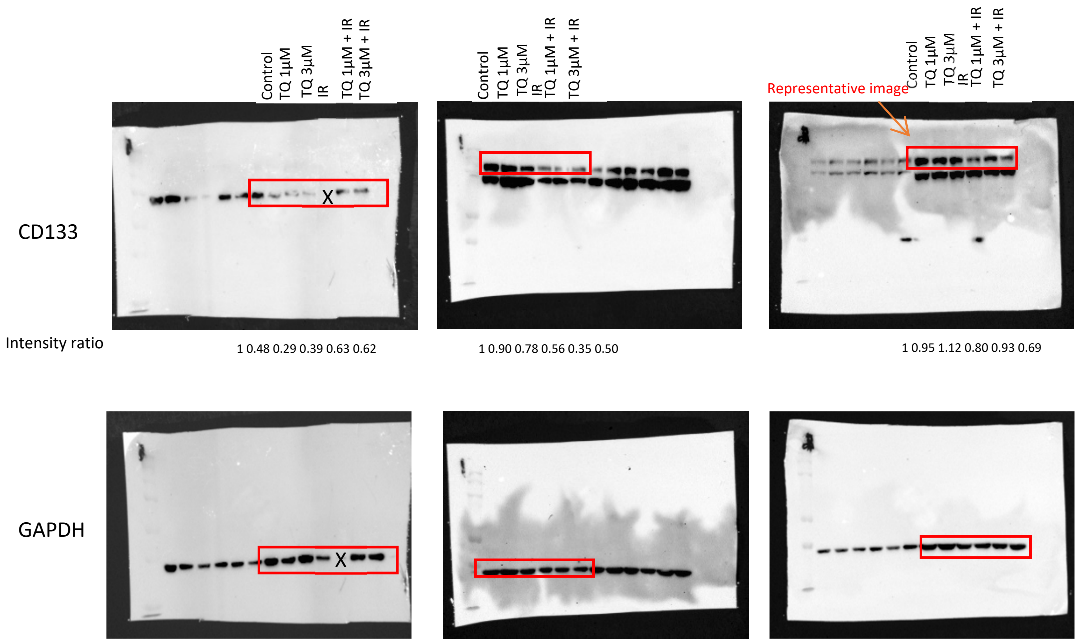

Figure S11. Whole western blot membranes for HCT116 (3D) (Figure 5c, left panel).

3D HT29 p53

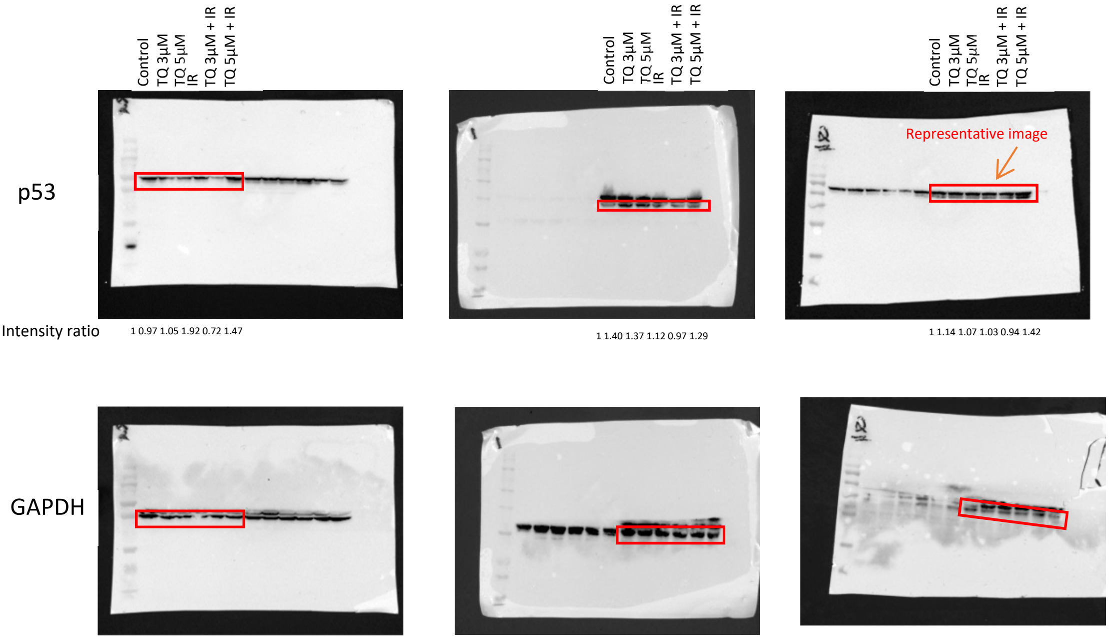

3D HT29 p21

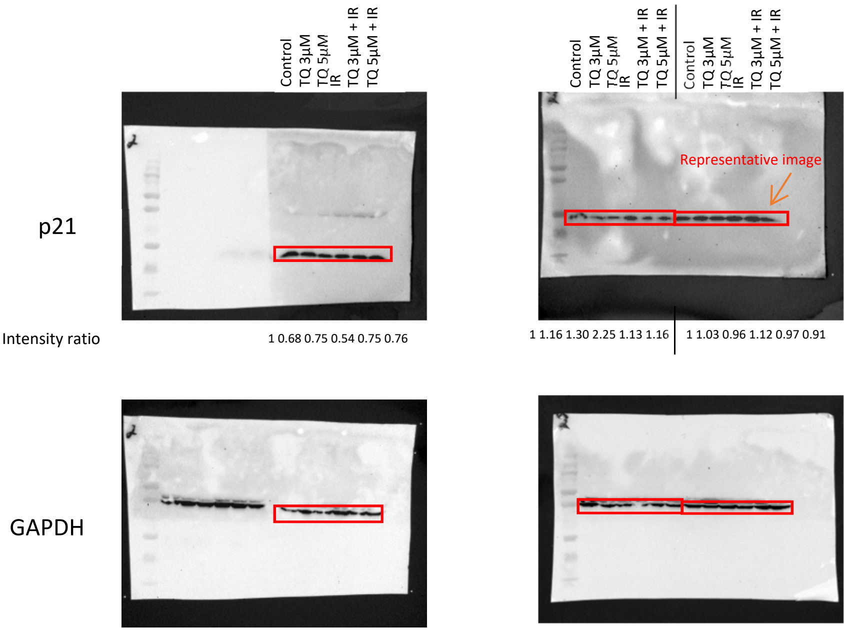

3D HT29 NF-κB

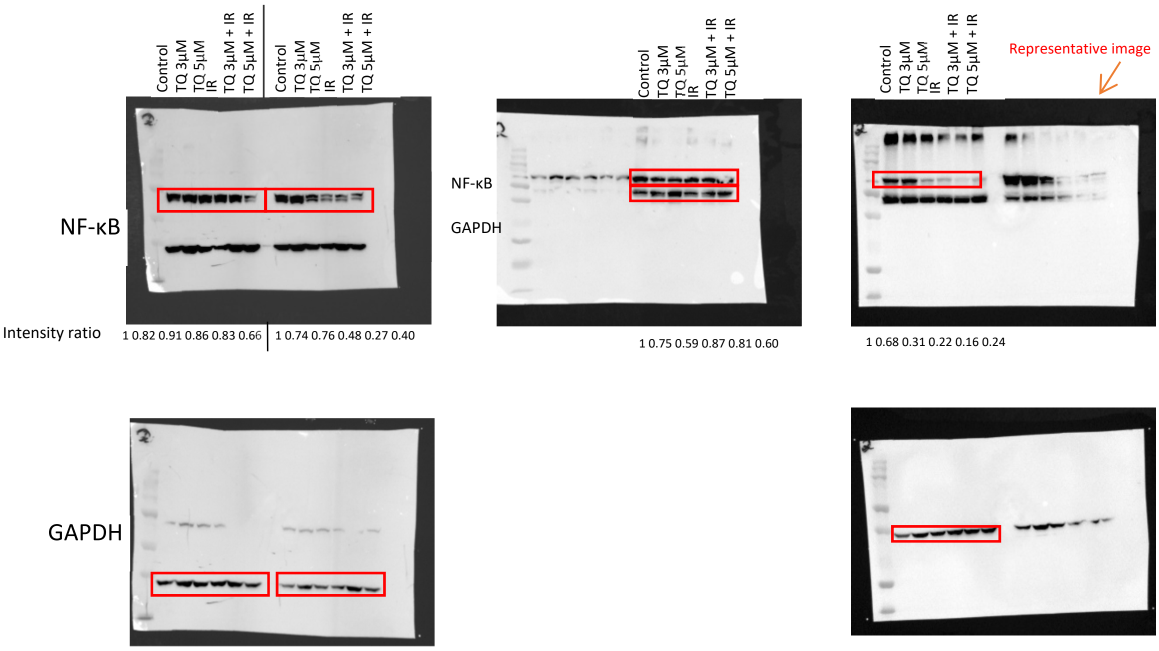

3D HT29 β catenin

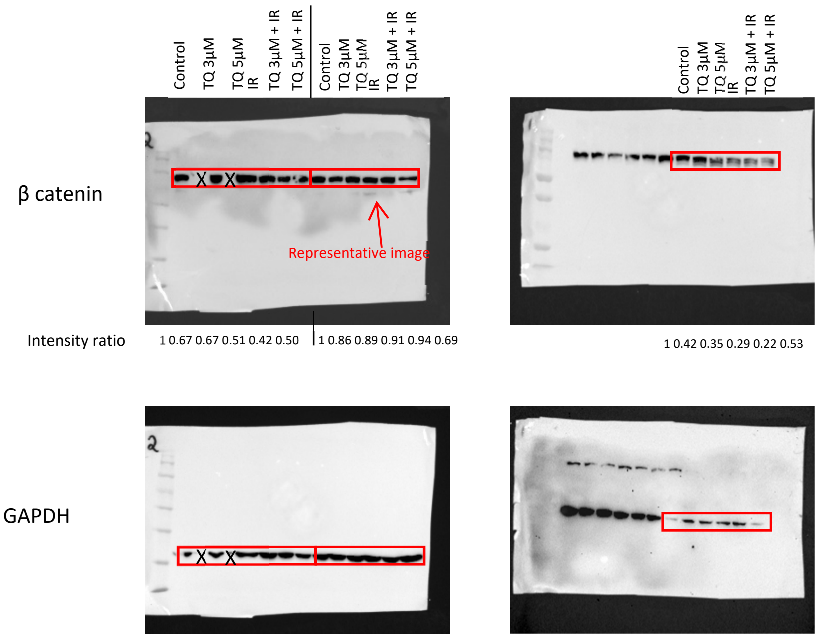

3D HT29 CD133

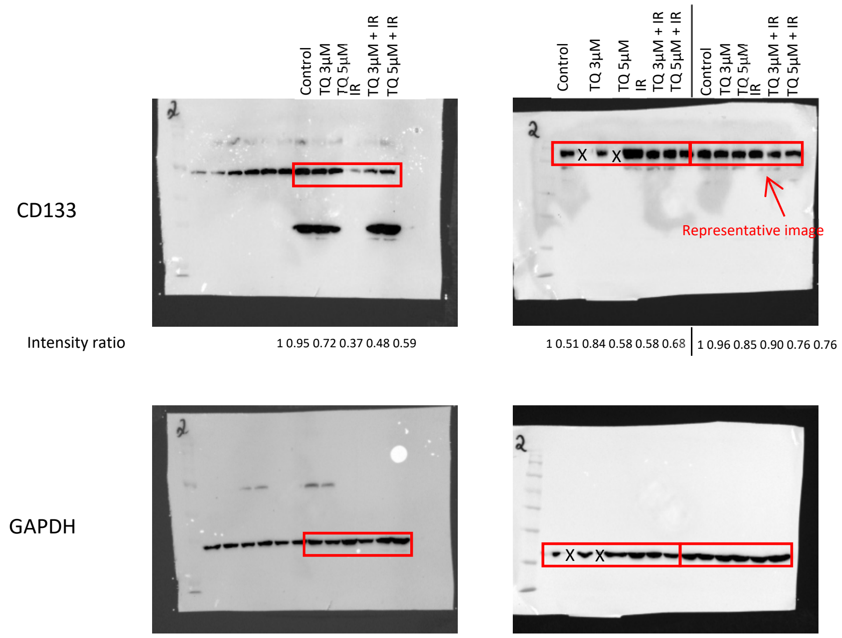

Figure S12. Whole western blot membranes for HT29 (3D) (Figure 5c, right panel).

Table S1. List of primary and secondary antibodies used in immunofluorescent staining.

Antibody Name	Species	Dilution	Catalog Number	Company
		**Primary antibodies**		
γH2AX	Rabbit	1:250	# 9718S	Cell signaling
p-ATM	Mouse	1:200	Sc-47739	Santa Cruz
p-ATR	Rabbit	1:200	sc-109912	Santa Cruz
p-mTOR	Rabbit	1:200	# 2971S	Cell signaling
MEK	Rabbit	1:200	# 9126S	Cell signaling
CD44	Mouse	1:100	sc-7297	Santa Cruz
CK8	Mouse	1:200	904801	Biolegend
CK19	Rabbit	1:200	ab15463	Abcam
		**Secondary antibodies**		
Alexa fluoro 488	Goat anti-mouse	1:400	A-28175	Invitrogen
Alexa fluoro 568	Goat anti-rabbit	1:200	A-11011	Invitrogen
Phalloidin		1:200	R415	Invitrogen

Table S2. List of primary and secondary antibodies used in western blot experiments.

Antibody Name	Species	Dilution	Catalog Number	Company
		Primary Antibodies		
β catenin	Mouse	1:200	sc-7963	Santa Cruz
CD133	Rabbit	1:500	# 64326S	Cell signaling
NF-κB p65	Rabbit	1:50	sc-372	Santa Cruz
p53	Rabbit	1:50	sc-6243	Santa Cruz
p21	Mouse	1:50	sc-6246	Santa Cruz
GAPDH-HRP (6C5)	Mouse	1:20,000	#MAB5476	Abnova
		Secondary Antibodies		
Goat anti-mouse	Goat	1:1000	sc-516102	Santa Cruz
Mouse anti-rabbit	Mouse	1:1000	sc-2357	Santa Cruz

Table S3. Significance between different groups stained for p-ATM, p-ATR, and γH2AX in HCT116 cells irradiated for 0 min, 10 min, or 24 h.

HCT116		
**Timepoints**	**Groups**	**Significance**
	**p-ATM**	
10 min	Control vs. TQ 30 μM + IR	* $p < 0.05$
	**p-ATR**	
10 min	Control vs. TQ 10 μM	* $p < 0.05$
	Control vs. IR	* $p < 0.05$
	Control vs. TQ 30 μM + IR	** $p < 0.01$
24 h	Control vs. TQ 30 μM + IR	** $p < 0.01$
	**γ-H2AX**	
0 min	Control vs. TQ 10 μM	* $p < 0.05$
	Control vs. TQ 30 μM	** $p < 0.01$
	Control vs. TQ 10 μM + IR	** $p < 0.01$
	Control vs. TQ 30 μM + IR	* $p < 0.05$
	IR vs. TQ 10 μM + IR	** $p < 0.01$
	IR vs. TQ 30 μM + IR	* $p < 0.05$
10 min	Control vs. TQ 10 μM	* $p < 0.05$
	Control vs. TQ 30 μM	* $p < 0.05$
	Control vs. IR	* $p < 0.05$
	Control vs. TQ 10 μM + IR	* $p < 0.05$
	Control vs. TQ 30 μM + IR	** $p < 0.01$
	TQ 30 μM vs. TQ 30μM + IR	* $p < 0.05$
24 h	Control vs. IR	* $p < 0.05$
	Control vs. TQ 10 μM + IR	* $p < 0.05$
	Control vs. TQ 30 μM + IR	** $p < 0.01$
	IR vs. TQ 30 μM + IR	** $p < 0.01$
	TQ 30 μM vs. TQ 30 μM + IR	* $p < 0.05$

Table S4. Significance between different groups stained for p-ATM, p-ATR, and γ H2AX in HT29 cells irradiated for 0 mins, 10 mins, or 24 hrs.

HT29		
**Timepoints**	**Groups**	**Significance**
	**p-ATM**	
0 min	Control vs. TQ 10 μM + IR	* $p < 0.05$
	Control vs. TQ 60 μM + IR	** $p < 0.01$
10 min	Control vs. TQ 60 μM	* $p < 0.05$
	Control vs. TQ 10 μM + IR	* $p < 0.05$
	Control vs. TQ 60 μM + IR	* $p < 0.05$
	**p-ATR**	
0 min	Control vs. TQ 10 μM	* $p < 0.05$
	Control vs. TQ 10 μM + IR	* $p < 0.05$
10 min	Control vs. TQ 60 μM	* $p < 0.05$
	Control vs. TQ 10 μM + IR	** $p < 0.01$
	Control vs. TQ 60 μM + IR	* $p < 0.05$
24 h	Control vs. TQ 10 μM	* $p < 0.05$
	Control vs. TQ 60 μM + IR	** $p < 0.01$
	**γ-H2AX**	
0 min	Control vs. TQ 10 μM	* $p < 0.05$
	Control vs. TQ 60 μM	*** $p < 0.001$
	Control vs. TQ 10 μM + IR	* $p < 0.05$
	Control vs. TQ 60 μM + IR	** $p < 0.01$
	IR vs. TQ 30 μM + IR	* $p < 0.05$
10 min	Control vs. IR	*** $p < 0.001$
	Control vs. TQ 10 μM + IR	** $p < 0.01$
	Control vs. TQ 60 μM + IR	** $p < 0.01$
	TQ 10μM vs. TQ 10 μM + IR	** $p < 0.01$
	TQ 60μM vs. TQ 60 μM + IR	* $p < 0.05$
24 h	Control vs. TQ 60 μM	** $p < 0.01$
	Control vs. IR	* $p < 0.05$
	Control vs. TQ 10 μM + IR	** $p < 0.01$
	Control vs. TQ 60 μM + IR	** $p < 0.01$
	IR vs. TQ 10 μM + IR	* $p < 0.05$
	IR vs. TQ 60 μM + IR	* $p < 0.05$
	TQ 10 μM vs. TQ 10 μM + IR	** $p < 0.01$
	TQ 60 μM vs. TQ 60 μM + IR	* $p < 0.05$

Table S5. Colorectal cancer patients′ clinical and histopathologic characteristics.

	Patient 1	Patient 2	Patient 3
Gender	Male	Female	Female
Age	62	55	61
BMI	29.7	25	21
Smoking	Yes	No	Yes
Chemotherapy preop	No	No	No
Radiation therapy preop	No	No	No
Location of tumor	Rectum	Sigmoid colon	Descending colon/ sigmoid colon
Type	Mucinous adenocarcinoma	Adenocarcinoma	Adenocarcinoma
T stage	pT2	pT2	pT3
N stage	pN0	pN0	pN0
M stage	N/A	N/A	N/A
Size of Tumor	6 cm	4.5 cm	2 cm
Grade	Not applicable: mucinous tumor	2: Moderately differentiated	2: Moderately differentiated
